# Toward resolving the RuO_2_/IrO_2_ oxygen evolution activity puzzle: critical roles of spin polarization and exchange–correlation functional accuracy

**DOI:** 10.1039/d6sc04703c

**Published:** 2026-10-09

**Authors:** Xiaokun Xie, Jiashun Wu, Zhenbin Wang

**Affiliations:** a Department of Materials Science and Engineering, City University of Hong Kong Hong Kong SAR 999077 China zwan22@cityu.edu.hk; b School of Energy and Environment, City University of Hong Kong Hong Kong SAR 999077 China

## Abstract

The oxygen evolution reaction (OER) activity ordering of RuO_2_ and IrO_2_ is well established experimentally, yet density functional theory calculations have persistently failed to reproduce this trend, undermining computational catalyst design. Here, we investigate this discrepancy by evaluating OER thermodynamic and kinetic activities of IrO_2_ and RuO_2_ using four exchange–correlation (XC) functionals (PBE, RPBE, r2SCAN, and MS2-rVV10) under spin-polarized and spin-unpolarized conditions. Spin polarization selectively stabilizes the O* intermediate on IrO_2_ while leaving RuO_2_ comparatively unaffected, and neglecting spin effects yields unphysically low kinetic barriers for IrO_2_. Among the four functionals, only MS2-rVV10 reproduces the experimentally observed activity trend in both thermodynamic and kinetic analyses. We trace these failures to a systematic correlation between adsorption-energy errors and deviations in the predicted activity trend. Notably, the OOH*–OH* scaling offset and theoretical limiting potential are themselves XC-functional dependent, cautioning against cross-functional comparison of overpotentials. Cross-code benchmarks rule out numerical implementation as the error source, while higher-rung functionals progressively overestimate Ru local magnetic moments in RuO_2_, introducing additional errors. This work establishes accurate exchange–correlation functionals and proper spin-polarization treatment as prerequisites for reliable OER catalyst design.

## Abstract 2

The oxygen evolution reaction (OER) activity ordering of RuO_2_ and IrO_2_ is well established experimentally, yet density functional theory calculations have persistently failed to reproduce this trend, undermining computational catalyst design.

## Graphical abstract

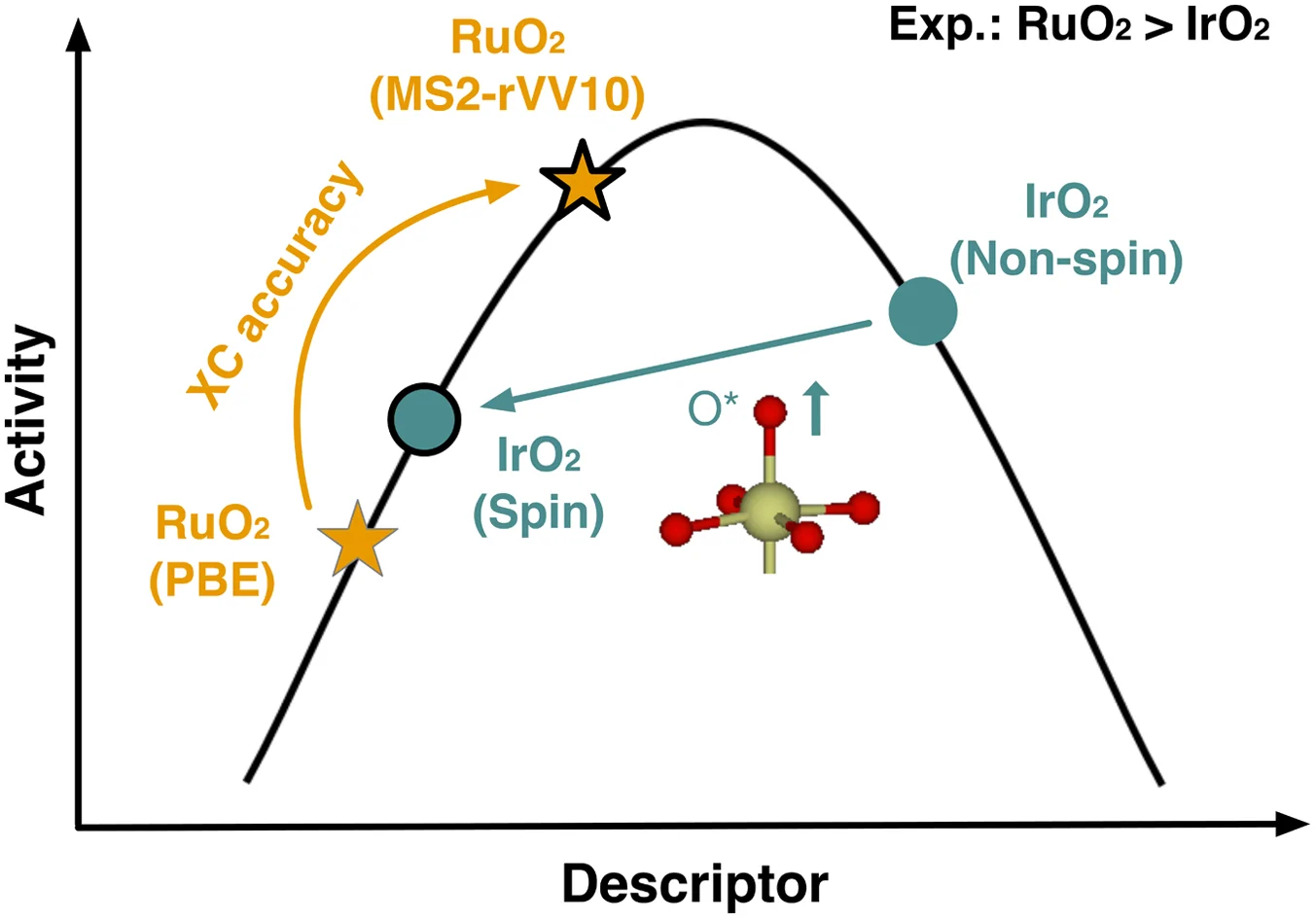

## Introduction

Electrocatalytic water splitting provides a sustainable pathway for green hydrogen production. The oxygen evolution reaction (OER) is the primary bottleneck limiting the overall efficiency of water electrolyzers.^1^ In acidic media, ruthenium dioxide (RuO_2_) and iridium dioxide (IrO_2_) are the two leading OER catalysts. It is well-established that RuO_2_ exhibits higher intrinsic OER activity than IrO_2_.^2^ Based on density functional theory (DFT)-calculated adsorption free energies, Man *et al.*^3^ established the well-known OER activity volcano, where RuO_2_ sits near the apex and IrO_2_ lies slightly down the left leg, close to the peak. This activity trend is consistent with experimental observations. However, reproducing this result has proven difficult in subsequent DFT calculations,^4–6^ as they consistently predict the opposite trend, with IrO_2_ significantly more active than RuO_2_, which contradicts experimental findings.

Several efforts have been made to identify the origin of this discrepancy. For example, Xu *et al.*^4^ applied the DFT+*U* method and found that inclusion of a Hubbard *U* term did not correct the erroneous activity trend. Briquet *et al.*^5^ examined the influence of different pseudopotentials within the PBE and RPBE frameworks: while the choice of pseudopotentials substantially affects the absolute values of the calculated overpotentials, it does not recover the experimental trend. Dickens *et al.*^7^ reported that defect sites on high-index RuO_2_ surfaces can yield lower overpotentials than the stable (110) surface, yet the predicted activity still remains below that of IrO_2_ (110). More recently, Divanis *et al.*^8^ proposed that RuO_2_ and IrO_2_ may follow different reaction mechanisms, which would render the calculated overpotential of RuO_2_ lower than that of IrO_2_. However, this hypothesis is at odds with the general experimental consensus that both catalysts proceed *via* the adsorbate evolution mechanism.^9,10^ Despite these efforts, the RuO_2_/IrO_2_ OER activity puzzle remains unresolved, hampering rational OER catalyst design.

Notably, the aforementioned studies share two common methodological features that have received little scrutiny. First, they rely almost exclusively on GGA-level functionals, leaving the role of exchange–correlation (XC) functional accuracy in the predicted activity trends largely unexplored. Second, several theoretical studies^3–5,7–11^ employed spin-unpolarized models, consistent with the negligible magnetic moments predicted at the PBE and RPBE levels. However, recent experiments have suggested that RuO_2_ may exhibit altermagnetic behavior,^11–13^ a unique magnetic phase in which the macroscopic magnetization vanishes while local spin polarizations persist at the atomic level. Because these local spin states may influence the surface energetics of adsorbed intermediates (*i.e.*, O*, OH*, OOH*), a systematic investigation of both XC functionals and spin-polarization treatments is warranted.

In this work, we systematically examine how XC functional choice and spin-polarization treatment affect DFT-predicted OER activity on IrO_2_ and RuO_2_. Using four functionals (PBE, RPBE, r2SCAN, and MS2-rVV10), we compare thermodynamic and kinetic descriptors under spin-polarized and spin-unpolarized conditions. We then assess how these choices influence the predicted activity ordering, the OOH*–OH* scaling relation, and the description of magnetic behavior in RuO_2_. Through this analysis, we aim to clarify the origins of the longstanding RuO_2_/IrO_2_ activity discrepancy and define the conditions required for more reliable computational screening of OER catalysts.

## Results

### Thermodynamic activity

To examine the spin and exchange–correlation (XC) effects, we calculated theoretical OER overpotentials (*η*_OER_) for IrO_2_ and RuO_2_ using the PBE, RPBE, r2SCAN, and MS2-rVV10 functionals, with and without spin polarization (Fig. 1). In spin-unpolarized calculations (Fig. 1a and b), the resulting *η*_OER_ values for IrO_2_ are 0.44 V (PBE), 0.40 V (RPBE), 0.66 V (r2SCAN), and 0.79 V (MS2-rVV10). The corresponding values for RuO_2_ are 0.89 V, 1.01 V, 0.83 V, and 0.52 V, respectively. While all four functionals place RuO_2_ on the strong-binding (left) leg of the volcano, their positioning of IrO_2_ is inconsistent: RPBE places it on the left leg, whereas PBE, r2SCAN, and MS2-rVV10 position it on the weak-binding (right) leg.

**Fig. 1:**
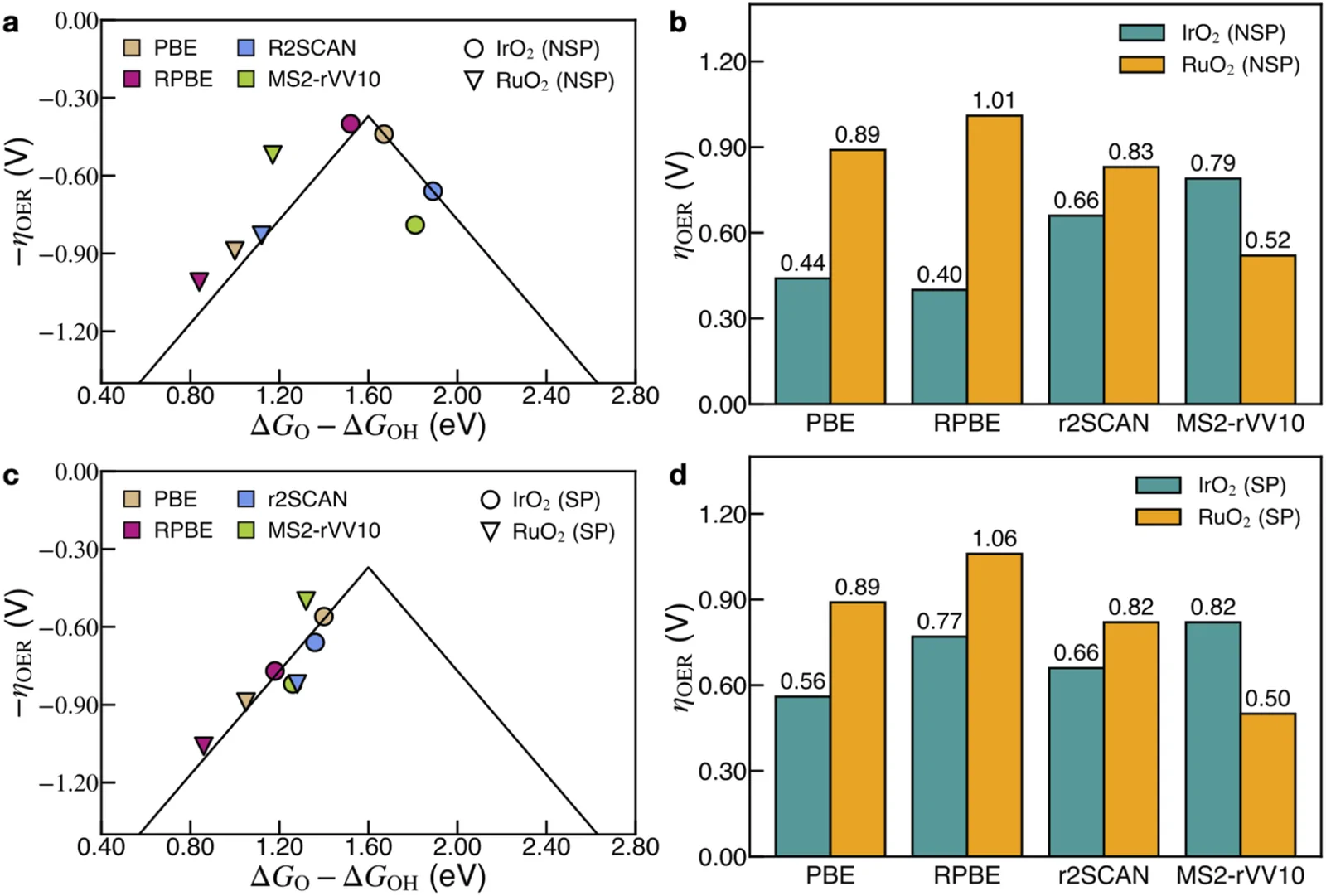
OER activity volcanoes and theoretical overpotentials for IrO_2_ and RuO_2_. (a and c) Volcano plots from spin-unpolarized (NSP) and spin-polarized (SP) calculations, respectively. (b and d) Corresponding theoretical overpotentials. Raw data are provided in Table S1.

When spin polarization is included, the calculated *η*_OER_ values shift to 0.56 V (PBE), 0.77 V (RPBE), 0.66 V (r2SCAN), and 0.82 V (MS2-rVV10) for IrO_2_, and 0.89 V (PBE), 1.06 V (RPBE), 0.82 V (r2SCAN), and 0.50 V (MS2-rVV10) for RuO_2_. Under spin-polarized conditions, all four functionals consistently position both IrO_2_ and RuO_2_ on the left leg of the volcano (Fig. 1c), indicating that OOH* formation is the potential-determining step (PDS). This result is consistent with the general understanding that OOH* formation limits the OER on these oxides.^14^ Furthermore, regardless of spin treatment, MS2-rVV10 is the only functional that correctly reproduces the experimentally observed activity trend in which RuO_2_ outperforms IrO_2_; PBE, RPBE, and r2SCAN all fail to capture this ordering.

Fig. 2a shows that the effect of spin polarization on the *η*_OER_ of IrO_2_ is strongly functional dependent: 0.12 V for PBE, 0.37 V for RPBE, and ≤0.03 V for r2SCAN and MS2-rVV10. This behavior originates mainly from the O* intermediate, which exhibits a substantial magnetic moment on IrO_2_ (0.69–0.81 *µ*_B_, Table S2). In spin-unpolarized calculations, the unpaired electron is artificially quenched, leading to underestimated O* binding. In contrast, OH* and OOH* are much less affected, with energy shifts of at most 0.11 eV and 0.04 eV, respectively. Although spin polarization stabilizes O* on IrO_2_ for all functionals, the impact on *η*_OER_ varies. For RPBE, the 0.37 eV stabilization translates directly into a 0.37 V increase in *η*_OER_, whereas for PBE, r2SCAN, and MS2-rVV10, the net change is much smaller because spin-induced O* stabilization shifts IrO_2_ from the weak- to the strong-binding side of the OER volcano, changing the PDS and partially offsetting the energetic gain (Fig. 1a and c).

**Fig. 2:**
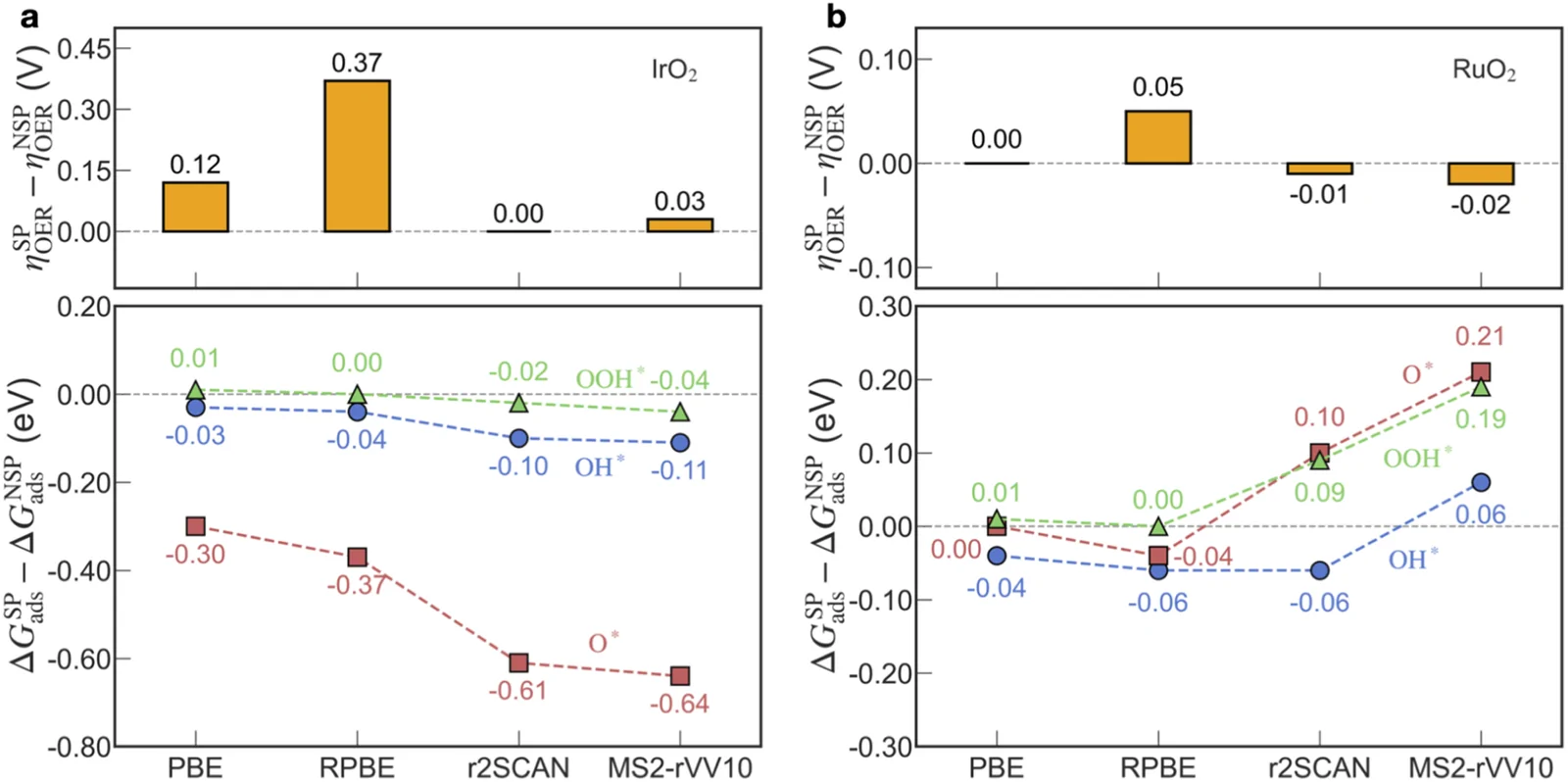
Effects of spin polarization on the calculated theoretical overpotentials (*η*) and adsorption free energies (Δ*G*) of OER intermediates on (a) IrO_2_ and (b) RuO_2_ across four functionals. The upper panels indicate the change in overpotential between spin-polarized (SP) and spin-unpolarized (NSP) calculations, Δ*η*^SP^_OER_ − *η*^NSP^_OER_. The lower panels show the corresponding difference in adsorption free energies between SP and NSP calculations, Δ*G*^SP^_ads_ − Δ*G*^NSP^_ads_, for OH* (blue circles), O* (red squares), and OOH* (green triangles). Dashed lines are added to guide the eye.

By comparison, spin polarization has little effect on the *η*_OER_ of RuO_2_ for any of the four functionals (Fig. 2b). This weak dependence is consistent with the small magnetic moments of the OER intermediates on RuO_2_ (<0.24 *µ*_B_, Table S2), which result in only minor changes in adsorption free energies. Even for MS2-rVV10, the largest shifts are only 0.06 eV for OH*, 0.21 eV for O*, and 0.19 eV for OOH*. These results show that spin polarization is essential for accurately describing IrO_2_, but has only a limited influence on RuO_2_.

To further assess the sensitivity of the predicted PDS to the choice of exchange–correlation (XC) functionals, free energy diagrams were calculated for both IrO_2_ and RuO_2_ with spin polarization. As shown in Fig. 3a, PBE, RPBE, and r2SCAN identify OOH* formation (eqn (3)) as the PDS on IrO_2_, whereas MS2-rVV10 identifies OOH* decomposition (eqn (4)). Notably, the identity of the limiting step on IrO_2_ remains unsettled in the literature. Most previous studies assign it to OOH* formation,^3,5,9,14^ whereas OOH* decomposition has also been proposed.^15^ Although MS2-rVV10 reproduces the experimental OER activity trend, its mean absolute error against the experimental adsorption-energy benchmark is 0.34 eV (*vide infra*), comparable to the free-energy margins that distinguish the two candidate steps. The divergence in Fig. 3a therefore highlights the sensitivity of the PDS assignment to the choice of XC functional, rather than providing sufficient evidence to establish the prediction of any one functional as definitive. By contrast, all four XC functionals consistently identify OOH* formation as the PDS for RuO_2_ (Fig. 3b), which is in agreement with previous kinetic analyses.^16,17^

**Fig. 3:**
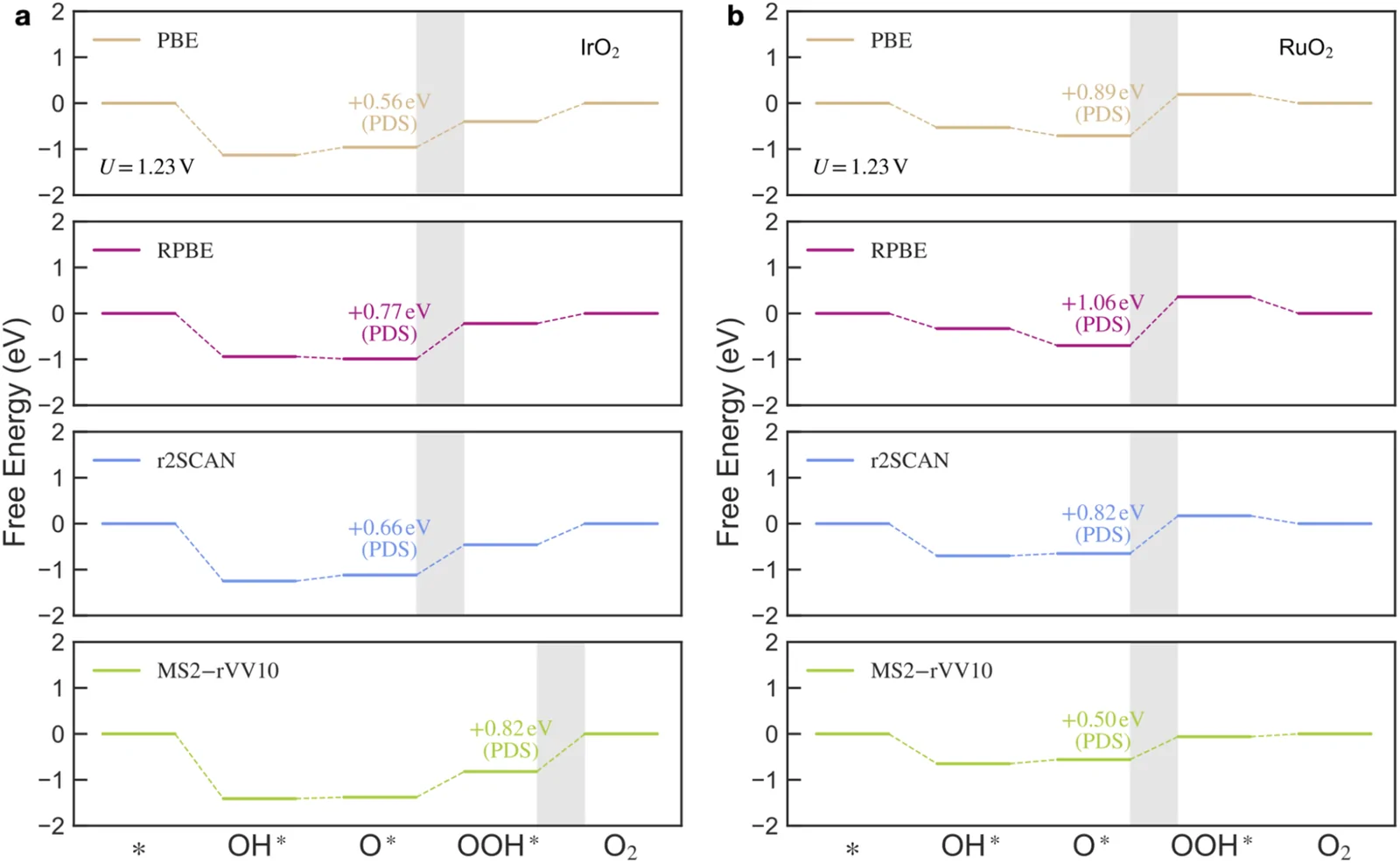
Free energy diagrams for the OER on (a) IrO_2_ and (b) RuO_2_ at 1.23 V *vs.* RHE, calculated with spin polarization. The grey shaded region indicates the potential-determining step (PDS).

The reason MS2-rVV10 predicts OOH* decomposition as the PDS on IrO_2_ is that it stabilizes OOH* strongly enough that O_2_ release from adsorbed OOH* becomes more energetically demanding than OOH* formation. Because Δ*G*_3_ = Δ*G*_OOH*_ − Δ*G*_O*_ and Δ*G*_4_ = 4.92 eV − Δ*G*_OOH*_, stabilizing OOH* simultaneously lowers Δ*G*_3_ and raises Δ*G*_4_. The two steps become energetically equivalent at the crossover value Δ*G*_OOH*_ = (4.92 eV + Δ*G*_O*_)/2. As shown in Table S3, MS2-rVV10 predicts Δ*G*_OOH*_ = 2.87 eV, which is 0.36–0.60 eV lower than the values obtained with the other three functionals and is the only value that lies below its corresponding crossover value. OOH* decomposition (Δ*G*_4_) is therefore the PDS. In contrast, the Δ*G*_OOH*_ values obtained with PBE, RPBE, and r2SCAN lie 0.08–0.27 eV above their respective crossover values, making OOH* formation (Δ*G*_3_) the PDS for these three functionals.

We note that a bridge-assisted AEM^8^ has been proposed to rationalize the OER activity ordering of IrO_2_ and RuO_2_. In this work, we follow the conventional AEM described in the Methods section; a detailed discussion of the bridge-assisted AEM is provided in the SI.

### Kinetic activity

Beyond thermodynamic activity, we assessed how the choice of XC functional affects the kinetic barrier for the corresponding PDS. As shown in Fig. 4a, the spin-polarized barriers obtained with PBE, RPBE, r2SCAN, and MS2-rVV10 are 0.43, 0.77, 0.58, and 0.77 eV for IrO_2_, and 0.74, 1.03, 0.69, and 0.62 eV for RuO_2_, respectively. The corresponding transition-state structures are shown in Fig. S1 and S2. For OOH* formation, an explicit H_2_O molecule approaches the reacting coordinatively unstaturated site (CUS) O* at the transition state, leading to O–O bond formation, while one proton is transferred to a neighboring CUS oxygen to form an adjacent OH*. The O–O bond lengths at the transition state are 1.77–1.89 Å on IrO_2_ and 1.80–1.86 Å on RuO_2_. Only MS2-rVV10 predicts a higher barrier on IrO_2_ than on RuO_2_, consistent with both our thermodynamic prediction and the experimental activity ordering. The difference in kinetic barriers between the two oxides exhibits strong functional dependence, ranging from 0.31 eV (PBE) and 0.26 eV (RPBE) to 0.11 eV (r2SCAN) and −0.15 eV (MS2-rVV10). Since OOH* formation is widely regarded as the limiting step for both IrO_2_ and RuO_2_, we also calculated the MS2-rVV10 barrier for OOH* formation on IrO_2_, allowing a direct comparison of the same elementary step on both surfaces. The resulting barrier is lower than that on RuO_2_, in contradiction with the experimental activity ordering (Fig. S3).

**Fig. 4:**
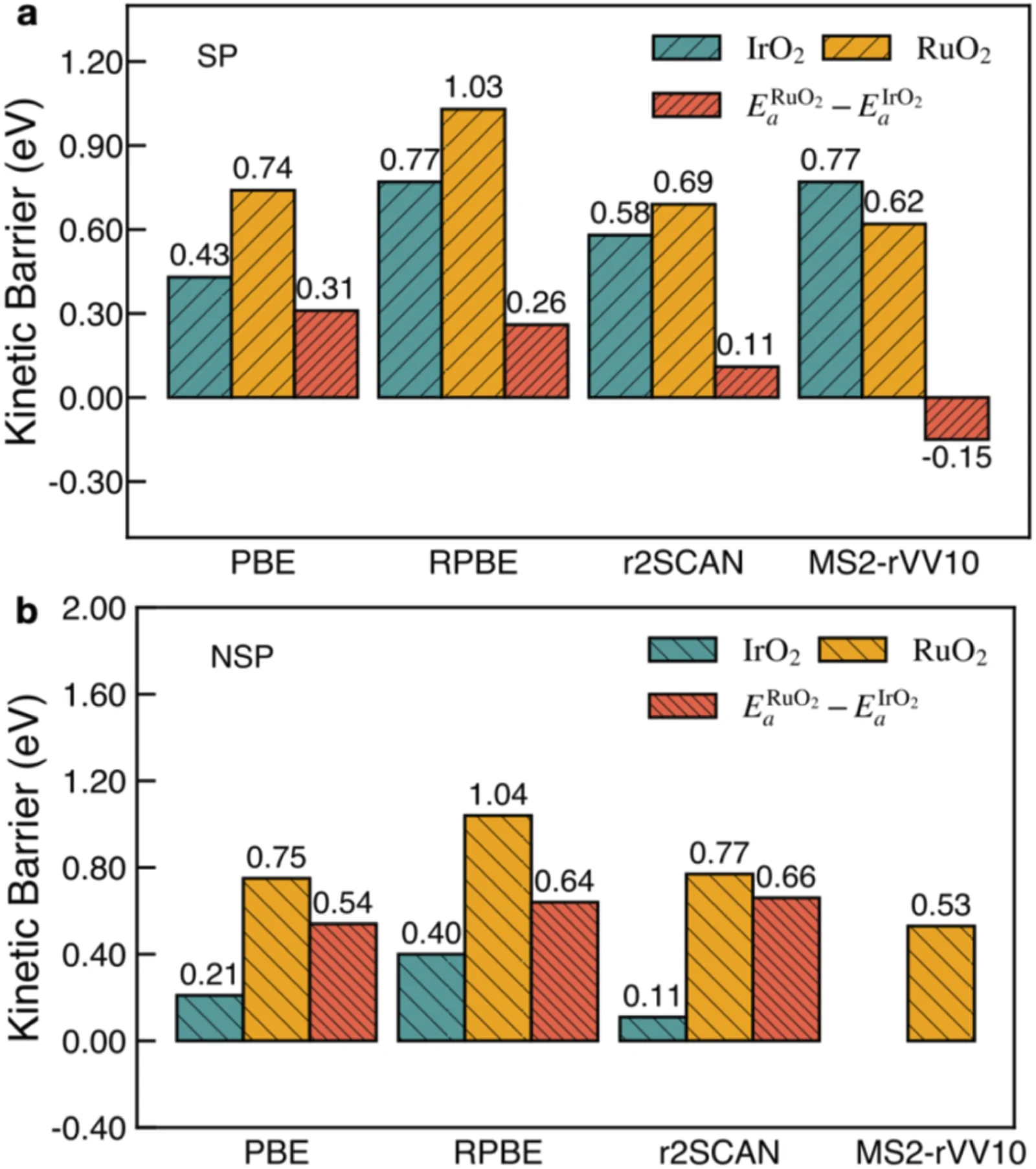
Kinetic barriers (*E*_a_) for the elementary step identified as the potential-determining step (PDS) by each XC functional on IrO_2_ and RuO_2_, calculated (a) with spin polarization (SP) and (b) without spin polarization (NSP). On IrO_2_, PBE, RPBE, and r2SCAN identify OOH* formation as the PDS, whereas MS2-rVV10 identifies OOH* decomposition; on RuO_2_, all four functionals identify OOH* formation. No barrier is reported for spin-unpolarized MS2-rVV10 on IrO_2_ (no transition state identified).

To further probe the role of spin effects, we also performed spin-unpolarized calculations. As shown in Fig. 4b, the barriers for IrO_2_ decrease dramatically to 0.21 (PBE), 0.40 (RPBE), and 0.11 (r2SCAN), which are unphysically low for OER. The corresponding spin-unpolarized MS2-rVV10 barrier is not reported because a transition state for OOH* decomposition on IrO_2_ could not be located. Instead, the spin-polarized calculation for the same elementary step converges to a well-defined transition state with a barrier of 0.77 eV. These contrasting results indicate that suppressing spin polarization can qualitatively distort the kinetic energy landscape on IrO_2_, leading to either unphysically low barriers or the failure to identify a transition state. Correspondingly, the barrier differences between IrO_2_ and RuO_2_ increase to 0.54, 0.64, and 0.66 eV, respectively. By contrast, RuO_2_ barriers are much less sensitive to spin polarization, with negligible changes for PBE/RPBE (0.01 eV) and only modest changes for r2SCAN/MS2-rVV10 (∼0.09 eV). This asymmetric sensitivity to spin effects is consistent with the thermodynamic analysis discussed previously and highlights that both the choice of XC functional and the proper treatment of spin polarization are essential for obtaining physically meaningful OER kinetic barriers, particularly for IrO_2_.

Solvation and applied-potential effects were further examined for OOH* formation on IrO_2_ and RuO_2_ at the PBE level (Fig. S4 and Table S4). Explicit-solvent *ab initio* metadynamics also predicts a markedly higher barrier on RuO_2_ than on IrO_2_, while constant-potential calculations at 1.60 V *vs.* RHE increase the barriers by less than 0.1 eV relative to the corresponding constant-charge values. Neither effect alters the predicted kinetic activity ordering, which places RuO_2_ substantially below IrO_2_ in contradiction with experiment.

### Origin of the OER activity discrepancy

We further investigated why the DFT-calculated thermodynamic and kinetic activities obtained with these functionals often fail to reproduce the experimental trend. Following Kothakonda *et al.*,^18^ who benchmarked the accuracy of various XC functionals for adsorption energies against experimental data, we plotted the reported functional uncertainty, defined as the deviation of the calculated adsorption energy from experiment, *δE*_ads_ (eqn (11)), as a function of the differences in DFT-calculated theoretical overpotentials and kinetic barriers between RuO_2_ and IrO_2_ (Fig. 5).

**Fig. 5:**
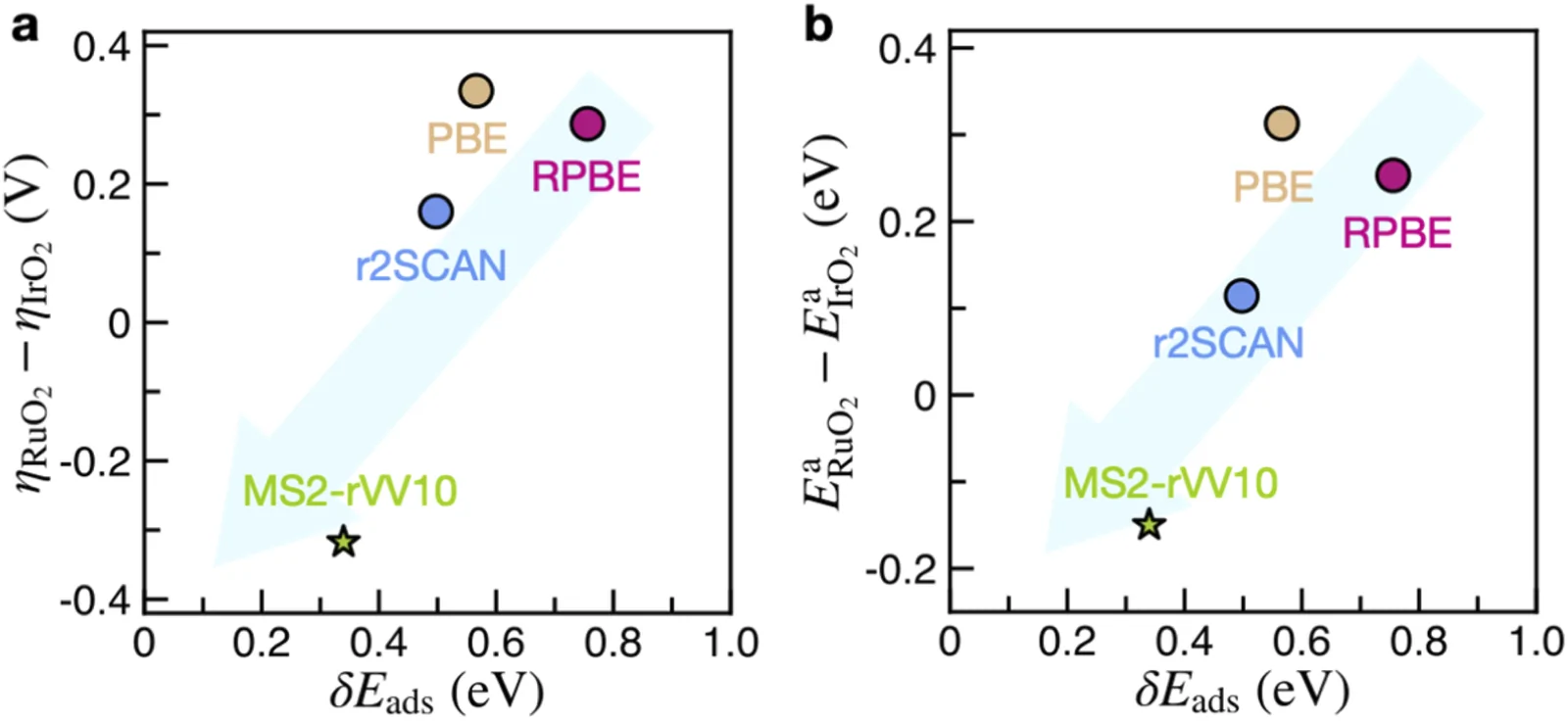
Differences in the calculated theoretical overpotentials (a) and kinetic barriers (b) between RuO_2_ and IrO_2_ plotted as a function of the adsorption-energy error (*δE*_ads_, eV). *δE*_ads_ is a composite descriptor of each XC functional's overall accuracy in predicting adsorption energies, benchmarked against experimental values.

A clear positive correlation is observed between *δE*_ads_ and the calculated activity differences. As *δE*_ads_ decreases, the differences in both theoretical overpotentials and kinetic barriers between RuO_2_ and IrO_2_ diminish, bringing the DFT predictions closer to the experimental activity trend. This suggests that functionals with higher accuracy in adsorption energies are also more capable of reproducing the experimental activity trend, namely that RuO_2_ is more active than IrO_2_. This finding underscores the critical role of XC functional accuracy in resolving the RuO_2_/IrO_2_ OER activity puzzle.

## Discussion

### XC-functional dependence of theoretical overpotentials

Fig. 6a presents the scaling relationship between Δ*G*_OH*_ and Δ*G*_OOH*_ calculated using the four XC functionals considered in this work, together with the nonlocal vdW-DF functional data reported by Briquet *et al.*^5^ We find that PBE, RPBE, r2SCAN, and MS2-rVV10 follow a scaling relationship with a nearly constant offset of 3.15 eV, which is close to the widely reported value of 3.20 eV.^3,19^ In contrast, the nonlocal vdW-DF functionals exhibit a significantly smaller offset of 2.71 eV. Previous studies have attributed such deviations to computational uncertainty.^20^ Here, we further emphasize that the shift is also strongly dependent on the choice of XC functional.

**Fig. 6:**
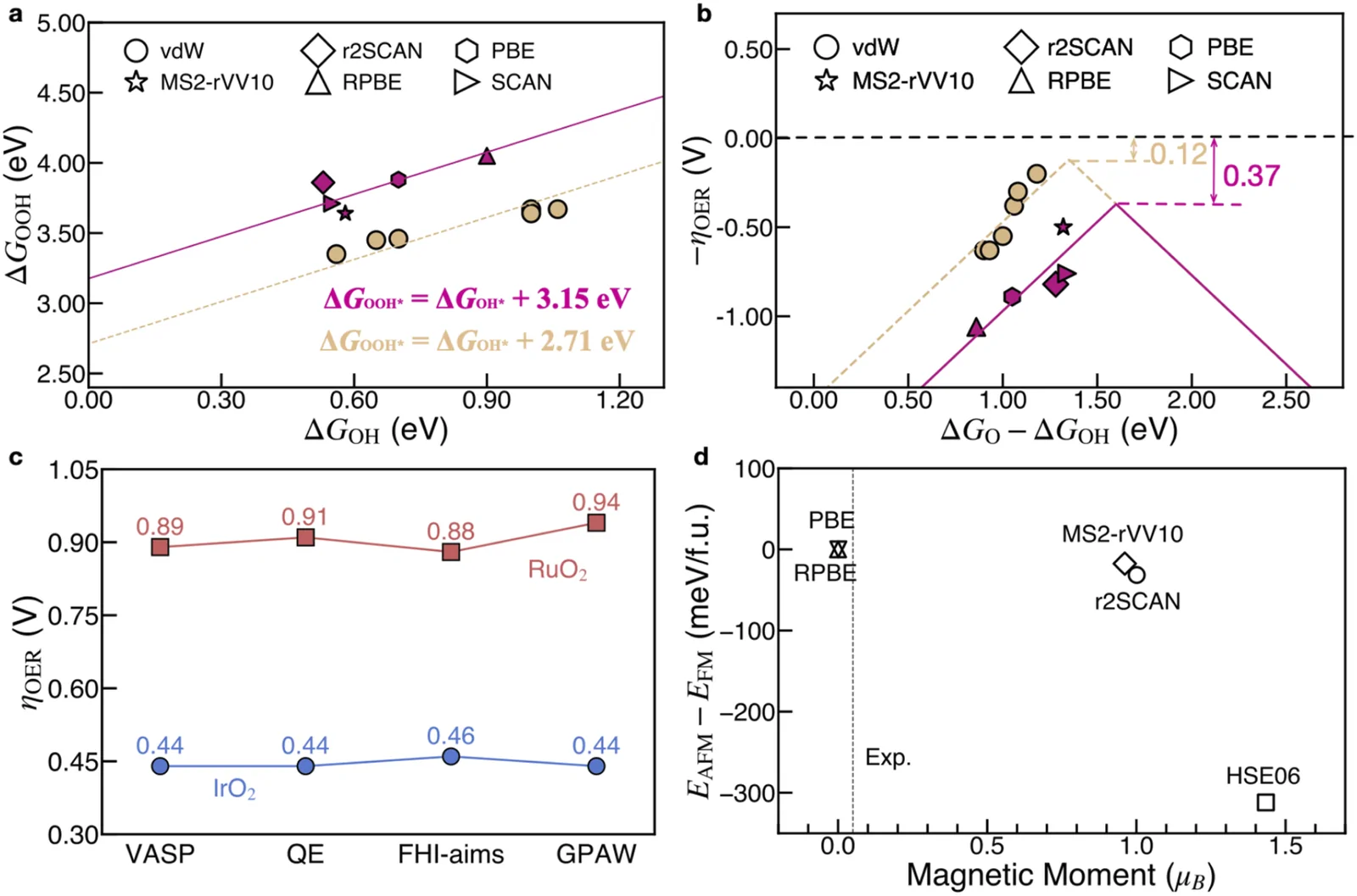
(a) Scaling relation between the adsorption free energies of OH* and OOH*. (b) OER activity volcano for RuO_2_ calculated using various functionals. The vdW data, calculated using vdW-DF,^21^ vdW-DF2 (ref. 22) and optPBE-vdW^23^ are taken from ref. 5. (c) Theoretical overpotentials calculated with the PBE functional across different DFT codes. (d) Energy difference between antiferromagnetic (AFM) and ferromagnetic (FM) configurations as a function of Ru magnetic moment in bulk RuO_2_ for different functionals. The Ru magnetic moment is zero in the FM state; the experimental value is indicated by a vertical dashed line.

Fig. 6b shows the theoretical OER volcano plots constructed from these two different scaling relationships. The corresponding theoretical limiting over potential changes substantially, from 0.37 V for a scaling offset of 3.20 eV to 0.12 V for an offset of 2.71 eV. This result clearly shows that the theoretical limiting potential is itself XC-functional-dependent. We further note that some studies have used nonlocal vdW-DF functionals to report theoretical overpotentials lower than 0.37 V and interpreted these values as evidence for breaking the conventional 3.20 eV scaling relationship. Our analysis suggests that such comparisons should be made with caution, because theoretical overpotentials reported in different studies are only directly comparable when they are evaluated within the same scaling framework.

It is also important to distinguish between calculated theoretical overpotentials and experimentally measured overpotentials. Experimental overpotentials depend on the chosen current density and therefore are not uniquely defined, even though the value at 10 mA cm^−2^ is commonly used as a benchmark.^24,25^ By contrast, the calculated theoretical overpotential is a thermodynamic descriptor derived from adsorption free energies. It therefore should not be directly compared with experimental overpotentials, which reflect both thermodynamic and kinetic contributions. As noted above, the theoretical overpotential is most appropriately used for predicting catalytic activity trends, often in conjunction with the BEP principle, and should be regarded as a descriptor for comparing relative activities rather than as a quantity directly equivalent to experimentally measured overpotentials.

### Sources of error in DFT-predicted OER activity trends

Briquet *et al.*^5^ previously reported that the calculated theoretical overpotential (*η*_OER_) can vary by as much as 0.33 V for IrO_2_ and 0.81 V for RuO_2_ across different DFT codes and ion-electron treatments, highlighting the strong influence of computational setup on OER predictions. Here, we revisited this issue by calculating *η*_OER_ for both IrO_2_ and RuO_2_ using three widely used plane-wave DFT codes, VASP, Quantum ESPRESSO, and GPAW, together with the all-electron code FHI-aims (Fig. 6c and Table S5). In our calculations, the variation in *η*_OER_ across these codes is only 0.02 V for IrO_2_ and 0.06 V for RuO_2_, indicating excellent consistency in the computed energetics. This observation is in line with the benchmark study of Lejaeghere *et al.*,^26^ which showed that recent, properly converged DFT codes yield highly reproducible results for PBE equations of state of elemental solids.

Besides the intrinsic errors of XC functionals in adsorption energies and kinetic barriers, overestimation of the Ru local magnetic moment may contribute to the remaining discrepancies between DFT-predicted and experimental OER activity trends, particularly for kinetic barriers. As discussed above, the magnetic state of Ru in RuO_2_ remains under debate, with some studies describing it as nonmagnetic and others proposing an altermagnetic ground state. We therefore analyzed the calculated Ru local magnetic moment in bulk RuO_2_ together with the energy difference between the FM and AFM states for the four XC functionals considered here, as well as for the hybrid functional HSE06, as shown in Fig. 6d. PBE and RPBE do not energetically distinguish the FM and AFM states, whereas r2SCAN, MS2-rVV10, and HSE06 predict the AFM state to be the ground state. The corresponding Ru local magnetic moments are 0, 0, 1.00, 0.96, and 1.43 µ_B_ for PBE, RPBE, r2SCAN, MS2-rVV10, and HSE06, respectively. In experimental studies reporting altermagnetism in RuO_2_, the measured Ru local moment is only ∼0.05 µ_B_, substantially smaller than the values predicted by the *meta*-GGA and HSE06 functionals. Moreover, within the set of functionals examined here, the predicted Ru moment generally increases from GGA to *meta*-GGA and hybrid functional, leading to progressively larger deviations from the experimentally reported small local moment. This trend is qualitatively consistent with the findings of Fu and Singh,^27^ who showed that *meta*-GGA and hybrid functionals can overestimate magnetism in itinerant transition metals by enhancing exchange splitting and favoring more localized, integer-like orbital occupations. Taken together, these results indicate that higher-rung functionals may overlocalize the Ru 4d electrons in RuO_2_, thereby overstabilizing magnetic solutions and introducing additional errors into the predicted OER energetics and activity ordering.

## Conclusion

By systematically varying XC functionals (PBE, RPBE, r2SCAN, and MS2-rVV10) and spin-polarization treatments, we have identified two main reasons why DFT has struggled to reproduce the experimental RuO_2_ > IrO_2_ OER activity ordering: limited XC-functional accuracy for surface adsorption energetics and the neglect of spin polarization. Spin polarization selectively stabilizes O* on IrO_2_ and is necessary to place both catalysts on the left leg of the OER volcano, consistent with prevailing mechanistic understanding for these oxides. Among the functionals tested, only MS2-rVV10 reproduces the experimentally observed activity ordering. The systematic correlation between adsorption-energy error and predicted activity difference further indicates that XC accuracy is a major factor governing whether the correct ordering is obtained.

More broadly, the XC-functional dependence of the OOH*–OH* scaling offset, and therefore of the theoretical limiting potential, shows that overpotentials reported with different functional frameworks are not directly comparable on a common scale. Future screening studies should therefore either use a single, consistently validated functional or explicitly report the scaling offset adopted, so that comparisons across studies remain meaningful.

Finally, the progressive overestimation of the Ru local magnetic moment by higher-rung functionals highlights a second challenge. Although such functionals are often expected to improve energetics, they can also overlocalize Ru 4d electrons in RuO_2_, overstabilize magnetic solutions, and introduce compensating errors. Resolving this combined requirement, accurate surface reaction energetics together with faithful local magnetic properties, may require machine-learned XC functionals or beyond-DFT-informed approaches trained against both chemisorption and magnetic reference data. Meeting this dual demand is therefore a key step toward reliable computational screening and rational design of OER catalysts.

## Methods

### Density functional theory calculations

Density functional theory (DFT) calculations were performed using the Vienna *Ab initio* Simulation Package (VASP) with the projector-augmented wave method.^28,29^ The PBE,^30^ RPBE,^31^ r2SCAN,^32^ and the MS2-rVV10 (ref. 18) functionals were used to describe exchange–correlation interactions. PBE and RPBE are widely used generalized gradient approximation (GGA) functionals, whereas r2SCAN and MS2-rVV10 are *meta*-GGA functionals. MS2-rVV10 has recently been reported to be one of the most accurate functionals for balancing physical and chemical adsorption energies against experimental data.^18^ The convergence criterion for the total energy was set to 1 × 10^−5^ eV for all calculations. Structural manipulations and input file generations were performed using the Atomic Simulation Environment (ASE).^33^

For bulk calculations, a plane-wave energy cutoff of 520 eV and a Gamma-centered 6 × 6 × 9 *k*-point grid was employed, with atomic forces converged to within 0.02 eV Å^−1^. The calculated lattice parameters for both rutile IrO_2_ and RuO_2_ are in good agreement with experimental values, with deviations of less than 1% for PBE, r2SCAN, and MS2-rVV10, and approximately 1.5% for RPBE (Table S6).

For surface calculations, a plane-wave energy cutoff of 450 eV was used, and atomic forces were converged to within 0.05 eV Å^−1^. The most stable (110) facets of rutile IrO_2_ and RuO_2_ were modeled using 2 × 1 slabs containing four atomic layers, with the bottom two layers fixed at their bulk positions. A vacuum spacing of 20 Å was applied perpendicular to the surface to prevent interactions between periodic images. Brillouin zone sampling was carried out using a Gamma-centered 3 × 3 × 1 *k*-point mesh. Spin-polarized calculations were performed using a ferromagnetic configuration for IrO_2_ and an antiferromagnetic configuration for RuO_2_.^34^ The magnetic ordering corresponding to the most stable surface structure of antiferromagnetic RuO_2_(110) is shown in Fig. S7.

### Thermodynamic activity calculations

The oxygen evolution reaction (OER) activity was evaluated at high O* coverage (Fig. S8), as surface Pourbaix diagram calculations identify this as the most stable surface configuration under typical OER working conditions (Fig. S9), consistent with previous studies.^3,5^ The OER was modeled using the adsorbate evolution mechanism (AEM),^3^ which proceeds through four concerted proton-coupled electron transfer steps:

1 H_2_O(l) + * → OH* + H^+^ + e^−^

2 OH* → O* + H^+^ + e^−^

3 O* + H_2_O(l) → OOH* + H^+^ + e^−^

4 OOH* → * + O_2_(g) + H^+^ + e^−^

where * denotes an active adsorption site on the surface. The reaction free energy changes of these four elementary steps (Δ*G*_1_, Δ*G*_2_, Δ*G*_3_, Δ*G*_4_) are described by the following equations:

5 Δ*G*_1_ = Δ*G*_OH*_

6 Δ*G*_2_ = Δ*G*_O*_ − Δ*G*_OH*_

7 Δ*G*_3_ = Δ*G*_OOH*_ − Δ*G*_O*_

8 Δ*G*_4_ = 4.92 eV − Δ*G*_OOH*_

For a thermochemically ideal catalyst, the reaction free energy for each of the four elementary steps is 1.23 eV. The step with the largest Gibbs free energy change is defined as the potential-determining step (PDS), and the theoretical overpotential (*η*_OER_) is given by:9
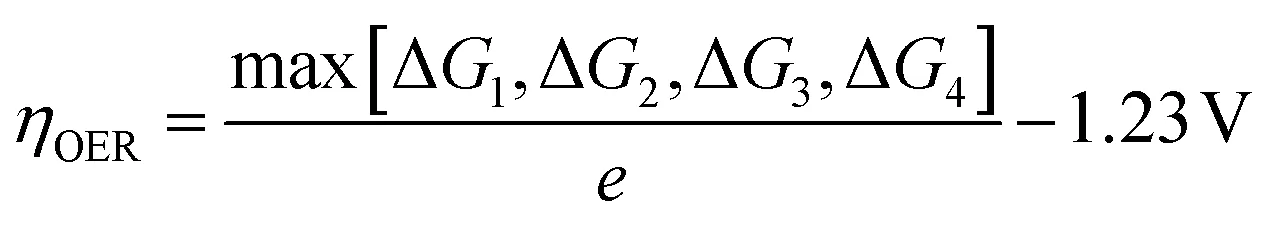


The Gibbs free energies of the adsorbed intermediates (Δ*G*_O_*n*_H_m_*_) were calculated from DFT-calculated total energies as follows:10
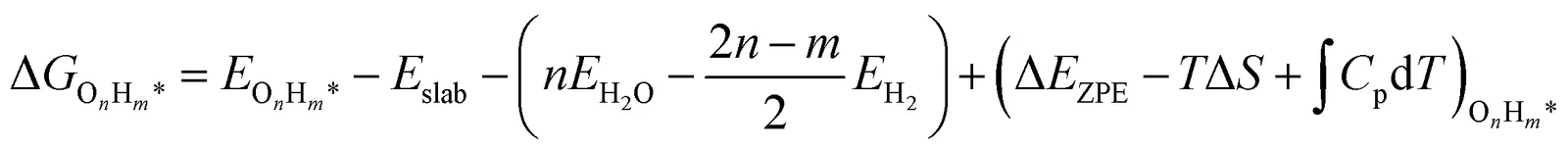
where Δ*E*_ZPE_ is the zero-point energy correction, *T*Δ*S* is the entropy contribution, and ∫*C*_p_d*T* is the integrated heat capacity from 0 to 300 K. The specific values for these corrections are listed in Table S7.

### Kinetic barrier calculation

For OOH* formation, an explicit H_2_O molecule was introduced at the surface to model O–O bond formation and concomitant proton transfer to a neighboring surface oxygen atom. For OOH* decomposition, deprotonation to O_2_* was followed by O_2_ desorption. Because the deprotonation barrier was negligible, only the O_2_ desorption step was considered in the transition-state analysis.^35^ The activation barriers were determined using the climbing-image nudged elastic band (CI-NEB) method.^36^ All identified transition states were verified through vibrational analysis, each exhibiting a single imaginary frequency.

The constant-potential effect on reaction kinetics was evaluated using VASPsol++ (ref. 37) at the PBE level with spin polarization. The absolute potential of the standard hydrogen electrode was set to −4.57 eV relative to the vacuum level, and the kinetic barrier was computed at an applied electrode potential of 1.60 V *vs.* RHE. The electrolyte was modeled as a 1 M aqueous solution with an ionic radius of 4 Å, treated within the linearized Poisson–Boltzmann scheme.

For metadynamics simulations,^38,39^ the model consisted of a 3 × 4 slab of the RuO_2_(110) or IrO_2_(110) surface, a 14 Å liquid-water layer (53 water molecules), and a 16 Å vacuum layer. Prior to metadynamics, the interfacial water structure was equilibrated by 8 ps of *ab initio* molecular dynamics. All simulations were performed in the NVT ensemble at 300 K using a Nosé–Hoover thermostat and a 1.0 fs time step, with the hydrogen mass set to 3.016 amu. Exchange–correlation effects were described by the PBE functional without spin polarization, which was neglected owing to the prohibitive cost of spin-polarized metadynamics sampling; van der Waals interactions were included *via* the DFT-D3 scheme with Becke–Johnson damping.^40^ A plane-wave cutoff of 450 eV was used, and the Brillouin zone was sampled at the *Γ*-point only. The collective variable (CV) was defined as the distance between the adsorbed oxygen (O*) and the oxygen of the attacking water molecule. Gaussian hills of 0.01 eV height and 0.1 Å width were deposited every 50 steps to bias the system across the activation barrier. To confine sampling to the targeted O–O coupling pathway, a Fermi-type biasing wall was applied to the O–H bond of the attacking water molecule, preventing premature O–H dissociation and diffusion of the molecule from the active site. As shown in Fig. S6, each simulation was continued until multiple forward and backward crossings between the O–O bonded (∼1.40 Å) and non-bonded (∼2.50 Å) states were observed, ensuring adequate sampling of the transition-state region (∼1.80 Å).

### Definition of the adsorption error descriptor

To provide a systematic measure of the overall accuracy of the exchange–correlation (XC) functionals in describing adsorption interactions, we introduced a composite error descriptor, *δE*_ads_. Consistent with the benchmarking strategy of Kothakonda *et al.*,^18^ the performance of each XC functional was evaluated in terms of the root-mean-square deviations (RMSDs) for chemisorption and physisorption energies, RMSD_chem_ and RMSD_phys_, respectively. The overall adsorption energy error *δE*_ads_ was then defined as the magnitude of the error vector in the two-dimensional space spanned by these two quantities:11
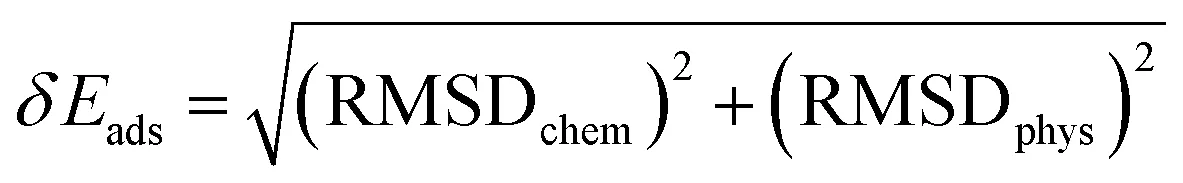


## Author contributions

Z. W. conceived the project and supervised the research. Z. W., X. X., and J. W. performed the calculations, analyzed the data, and drafted the manuscript. Z. W. revised the manuscript. All authors discussed the results and commented on the manuscript. X. X. thanks Dr Shuwei Li for his assistance in preparing the initial manuscript.

## Conflicts of interest

The authors declare no conflicts of interest.

## Supplementary Material

SC-OLF-D6SC04703C-s001

## Data Availability

All the data supporting this study are included in the article and its supplementary information (SI). Supplementary information: addition computational details, including structural optimization, metadynamics, and CI-NEB calculations; OOH* reaction geometries and kinetic barriers; antiferromagnetic ordering of RuO_2_(110); calculated surface Pourbaix diagram using different exchange correlation functionals; calculated adsorption free energies and magnetic moments of OER intermediates; constant potential and constant charge barrier comparisons; calculated adsorption energies from different DFT codes; calculated lattice constants of rutile IrO_2_ and RuO_2_; free energy corrections; and comparison of the conventional and bridge-assisted adsorbate evolution mechanisms. See DOI: https://doi.org/10.1039/d6sc04703c.
