# Supplementary material for: Toward resolving the RuO2/IrO2 oxygen evolution activity puzzle: critical roles of spin polarization and exchange–correlation functional accuracy
Source: Chem Sci. 2026 Oct 9. Online ahead of print. doi: 10.1039/d6sc04703c (PMC13673306; doi:10.1039/d6sc04703c)
Supplement: Supplementary file 1 [file SC-OLF-D6SC04703C-s001.pdf]

Supplementary Information (SI) for Chemical Science.
This journal is © The Royal Society of Chemistry

Supporting Information

Toward Resolving the RuO_2/IrO_2 Oxygen Evolution Activity Puzzle: Critical Roles of Spin Polarization and Exchange-Correlation Functional Accuracy

Xiaokun Xie[1†], Jiashun Wu[1†], Zhenbin Wang[1,2*]

[1]Department of Materials Science and Engineering, City University of Hong Kong, Hong Kong SAR, 999077, China

[2]School of Energy and Environment, City University of Hong Kong, Hong Kong SAR, 999077, China

[†]These authors contributed equally to this work.

E-mail: zwan22@cityu.edu.hk

Extended Methods

Additional density functional theory (DFT) calculations were performed with three codes: FHI-aims, which employs numerical atom-centered basis sets[1]; GPAW[2] ; and Quantum ESPRESSO[3], the latter two employing plane-wave basis sets. The Perdew–Burke–Ernzerhof (PBE) exchange–correlation functional[4] was employed in all calculations. The Brillouin zone was sampled with a Γ-centered 4×4×1 *k*-point mesh. A vacuum spacing of 20 Å was added along the surface normal, and a dipole correction was applied in the same direction. All structures were relaxed until the maximum force on any atom fell below 0.05 eV/Å.

For FHI-aims, "tight" numerical basis sets were used, and scalar-relativistic effects were included via the atomic zero-order regular approximation (ZORA). The self-consistent field (SCF) cycle was converged until the total energy, charge density, and sum of eigenvalues were accurate to within 1×10^{-6} eV, 1×10^{-5} e/Å^3, and 1×10^{-3} eV, respectively.

For GPAW, a plane-wave cutoff of 800 eV was used. The electronic occupation was treated with Fermi–Dirac smearing of 0.1 eV, and the total energy was converged to 1×10^{-5} eV.

For Quantum ESPRESSO, pseudopotentials from the SSSP Efficiency library[5] were adopted. The kinetic-energy cutoffs for the wavefunctions and charge density were set to 55 Ry and 440 Ry, respectively. Gaussian smearing of 0.1 eV was applied to the electronic occupations, and the electronic SCF loop was converged to 1×10^{-6} Ry.

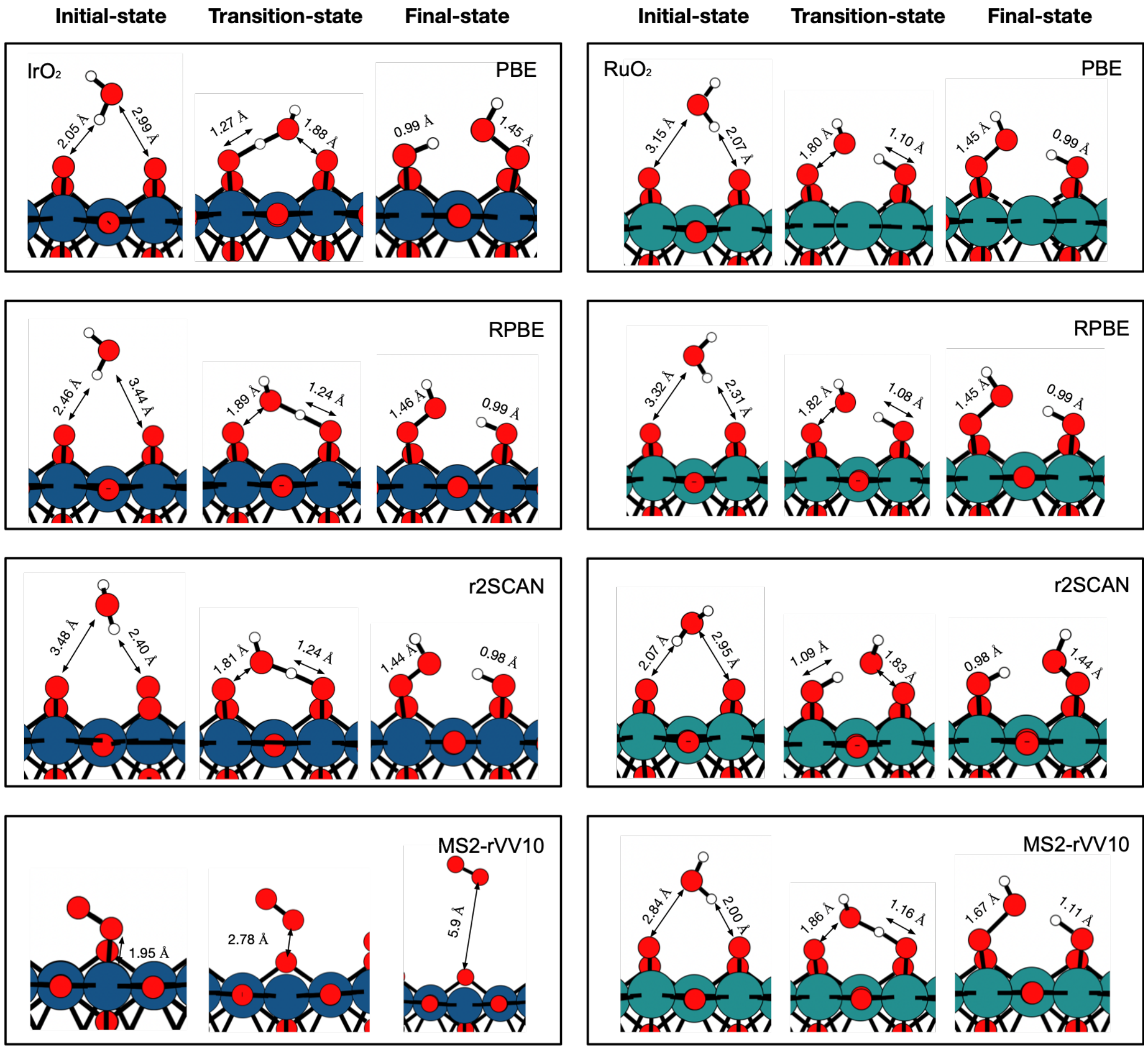

Figure S1. Representative geometries of the initial, transition, and final states of the elementary step identified as the potential-determining step (PDS) by each XC functional on IrO_2 and RuO_2, calculated with spin polarization. On IrO_2, PBE, RPBE, and r2SCAN identify OOH* formation as the PDS, whereas MS2-rVV10 identifies OOH* decomposition; on RuO_2, all four functionals identify OOH* formation. Blue, green, red, and white spheres represent Ir, Ru, O, and H atoms, respectively.

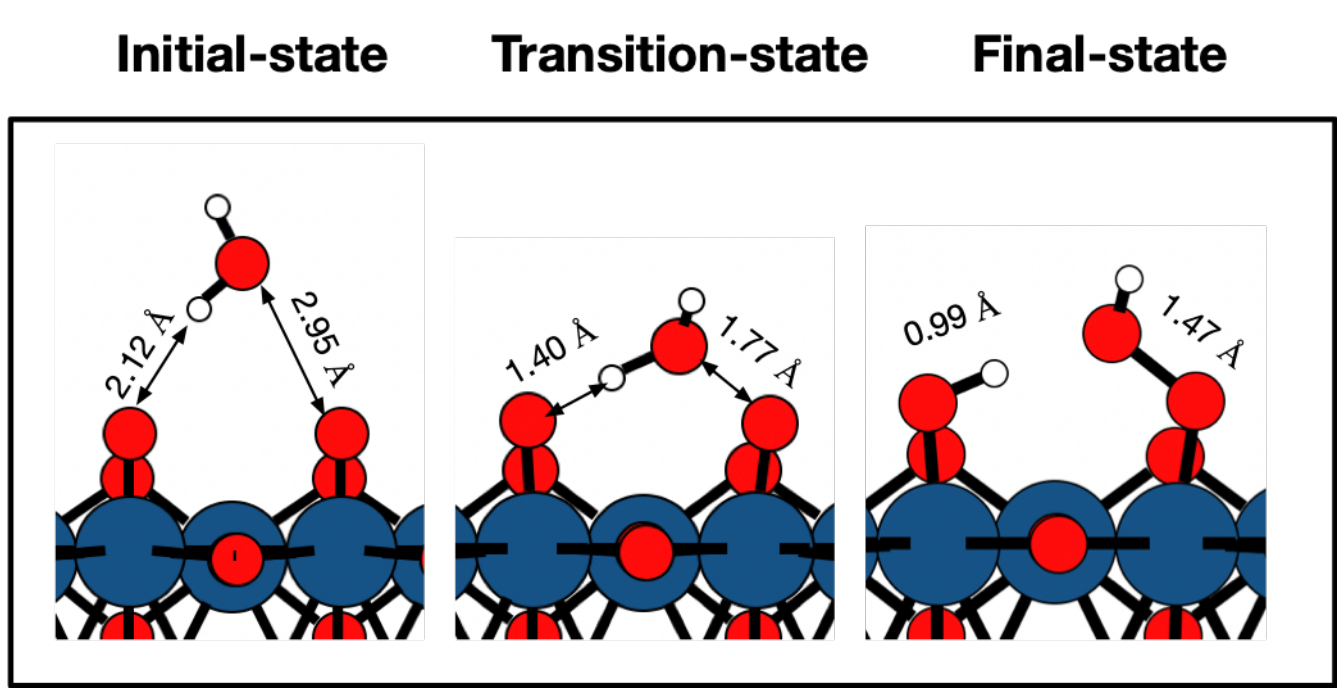

Figure S2. Representative geometries of the initial, transition, and final states for OOH* formation on IrO_2, calculated with MS2-rVV10 including spin polarization. Blue, red, and white spheres represent Ir, O, and H atoms, respectively.

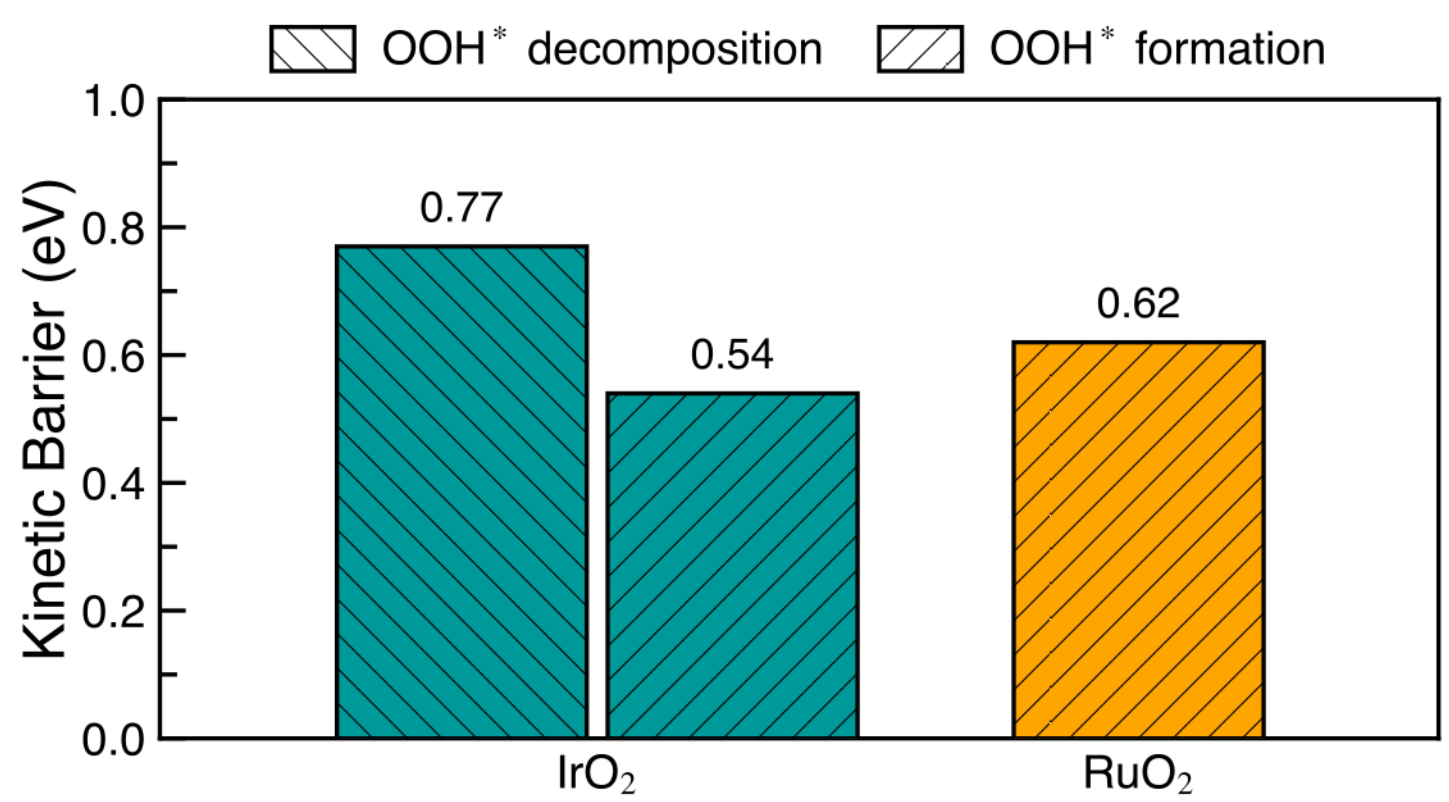

Figure S3. Kinetic barriers calculated with MS2-rVV10 for OOH* decomposition and formation on IrO_2 and for OOH* formation on RuO_2.

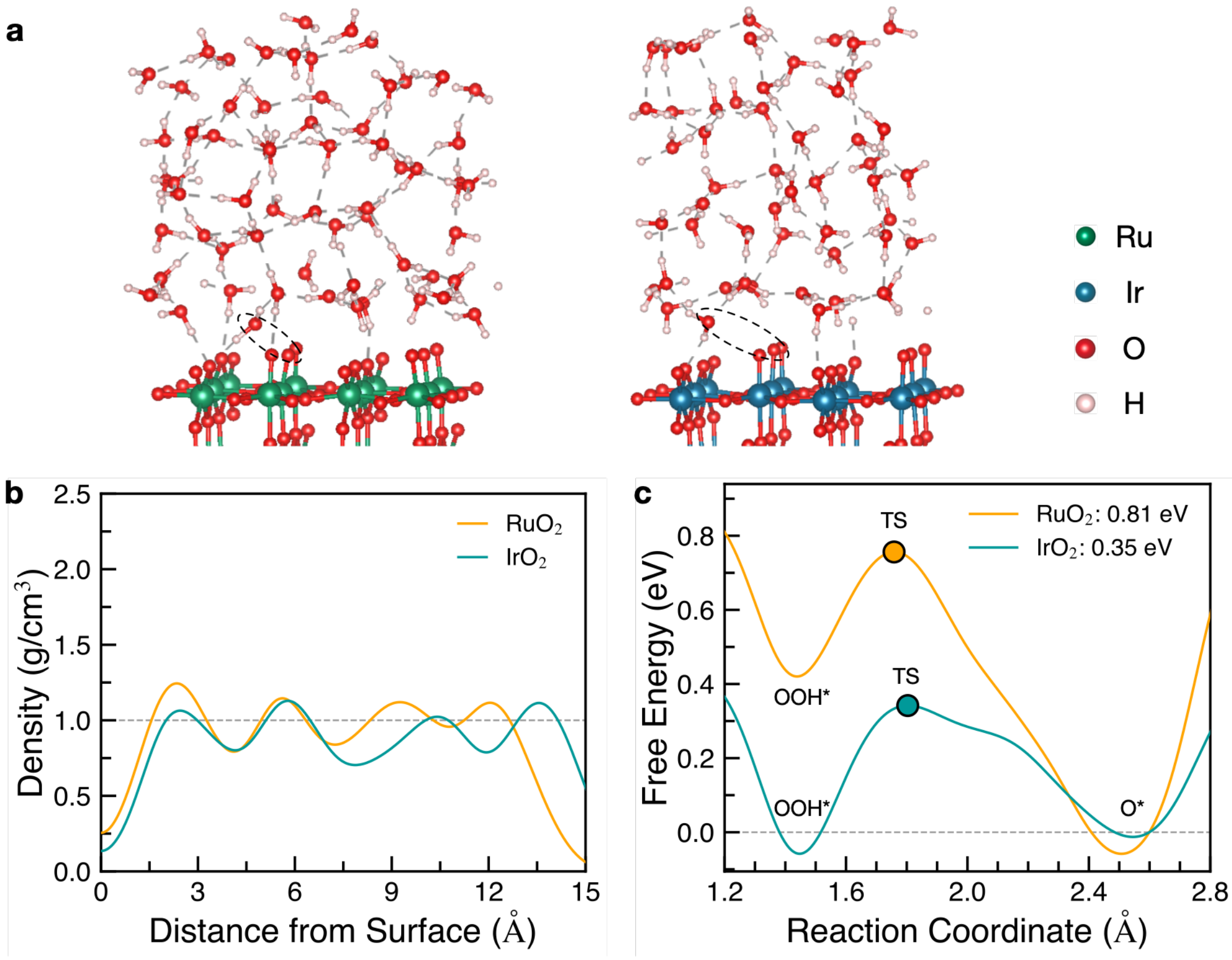

Figure S4. (a) Representative snapshots of the equilibrated oxide/water interface structure for RuO_2 and IrO_2. (b) Mass-density profiles of interfacial water along the surface normal, converging to ~1.0 g/cm^3 in the bulk region. (c) Calculated free-energy profiles for OOH* formation at 300 K, with the O*, transition state (TS), and OOH* states labeled.

To assess the influence of solvation on the OER kinetics, we further examined OOH* formation on the RuO_2(110) and IrO_2(110) surfaces using *ab initio* metadynamics with explicit solvation at the PBE-D3 level[6, 7]. Figure S4a shows representative snapshots of the equilibrated oxide/water interfaces. For both systems, the water mass-density profiles along the surface normal (z-axis) converge to 1.0 g/cm^3 in the bulk region (Figure S4b), confirming that the model is sufficiently large to recover bulk-like water behavior. As shown in Figure S4c, Figure S5 and Figure S6, the calculated activation barrier for IrO_2 is 0.35 eV, with an O–O bond length of 1.80 Å at the transition state, whereas RuO_2 exhibits a markedly higher barrier of 0.81 eV and a shorter O–O distance of 1.76 Å. In addition, we evaluated the constant-potential effect on the calculated kinetic barrier for OOH* formation at the PBE level. At an applied potential of 1.60 V vs RHE, the barrier is 0.52 eV for IrO_2 and 0.80 eV for RuO_2, only 0.09 and 0.06 eV higher than the corresponding constant-charge values (Table S4), respectively. Taken together, these results indicate that solvation and applied-potential effects on the predicted kinetic barriers are small and do not alter conclusions.

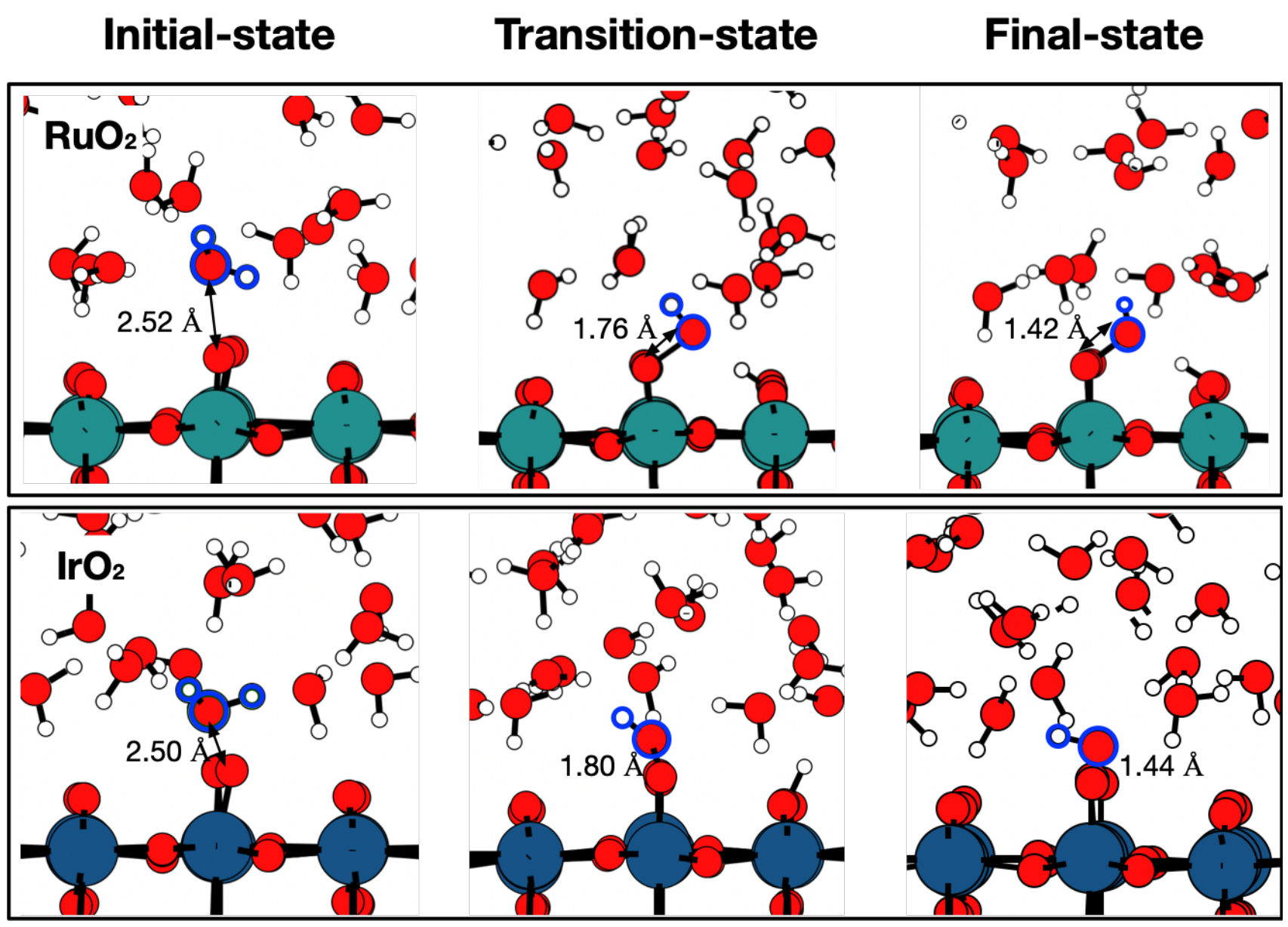

Figure S5. Representative interfacial geometries along OOH* formation on RuO_2 and IrO_2. Blue circles indicate the reacting oxygen species involved in O–O bond formation and proton transfer. Red, white, blue, and green spheres represent O, H, Ir, and Ru atoms, respectively.

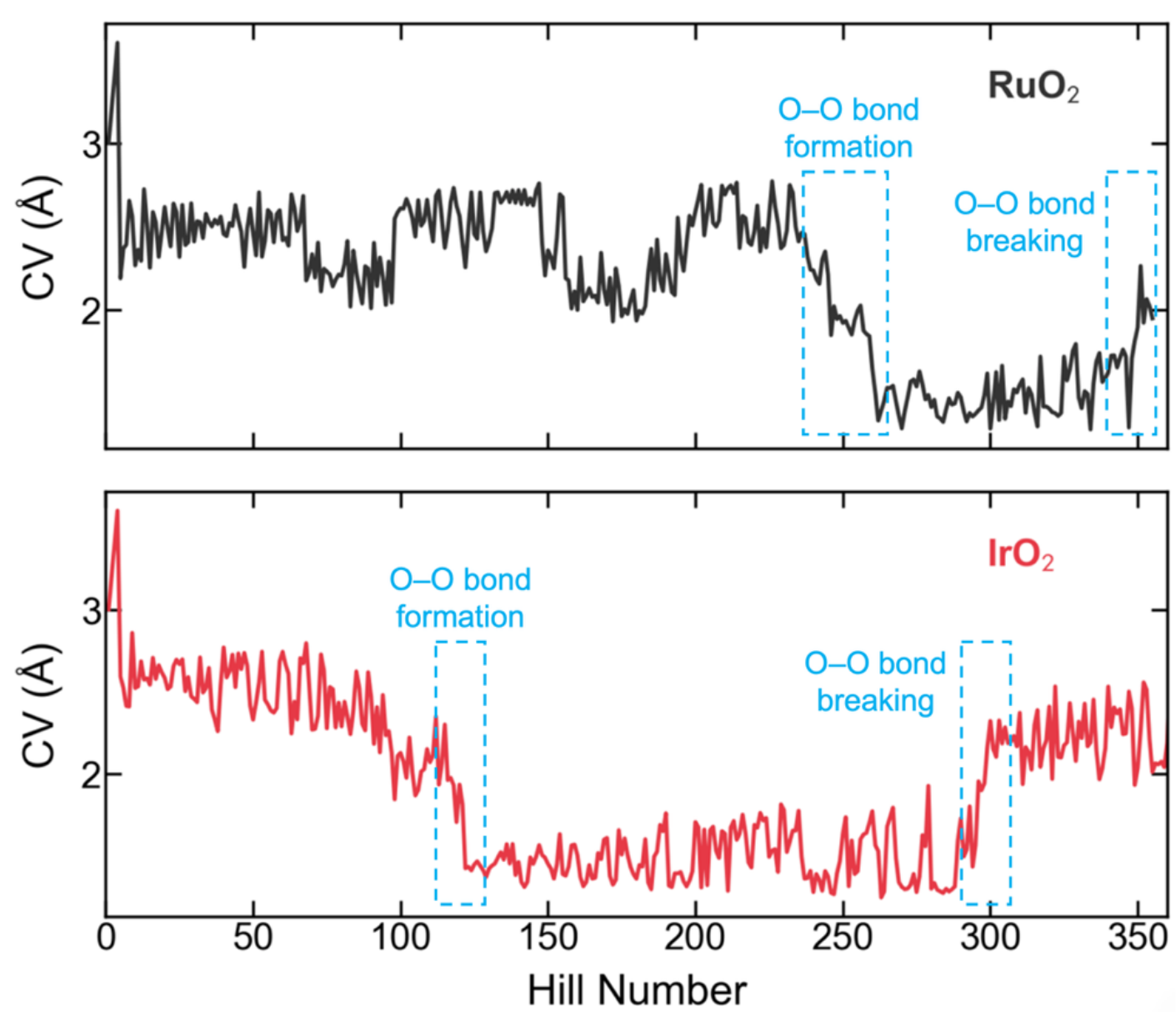

Figure S6. Collective variable (CV) trajectories under different Hill numbers.

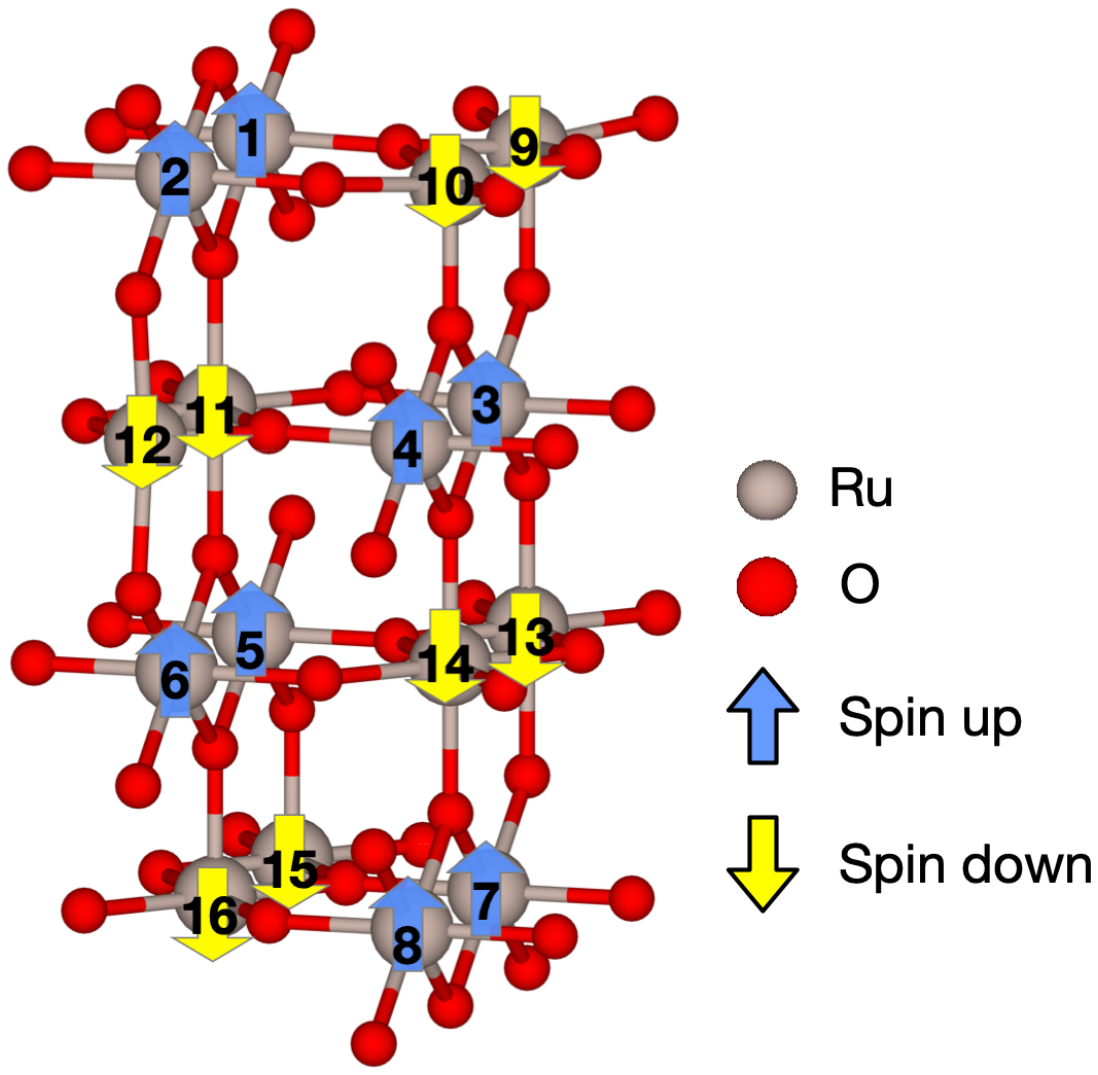

Atom index	Magnetic moment (μ_B)	Atom index	Magnetic moment (μ_B)
1	1.346	9	-0.906
2	1.346	10	-0.906
3	0.809	11	-0.809
4	0.809	12	-0.809
5	0.843	13	-0.790
6	0.843	14	-0.790
7	1.437	15	-0.772
8	1.437	16	-0.772

Figure S7. Magnetic orderings of Ru atoms on the antiferromagnetic RuO_2 (110) surface. The left panel illustrates the spin configuration, which was reported to be the most stable AFM configuration[8]. The right panel shows the corresponding local magnetic moments calculated using the MS2-rVV10 functional.

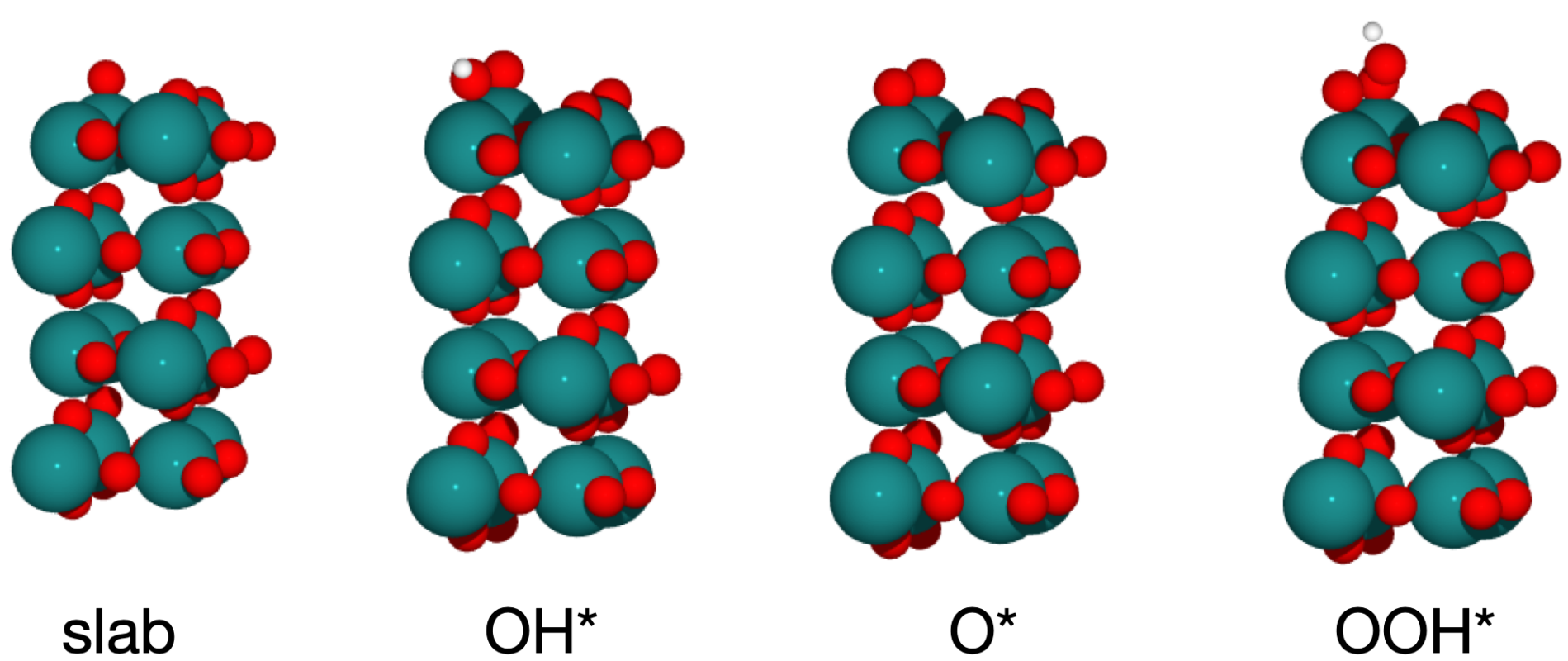

Figure S8. Schematic diagrams of the surface models used in the oxygen evolution reaction (OER) calculations.

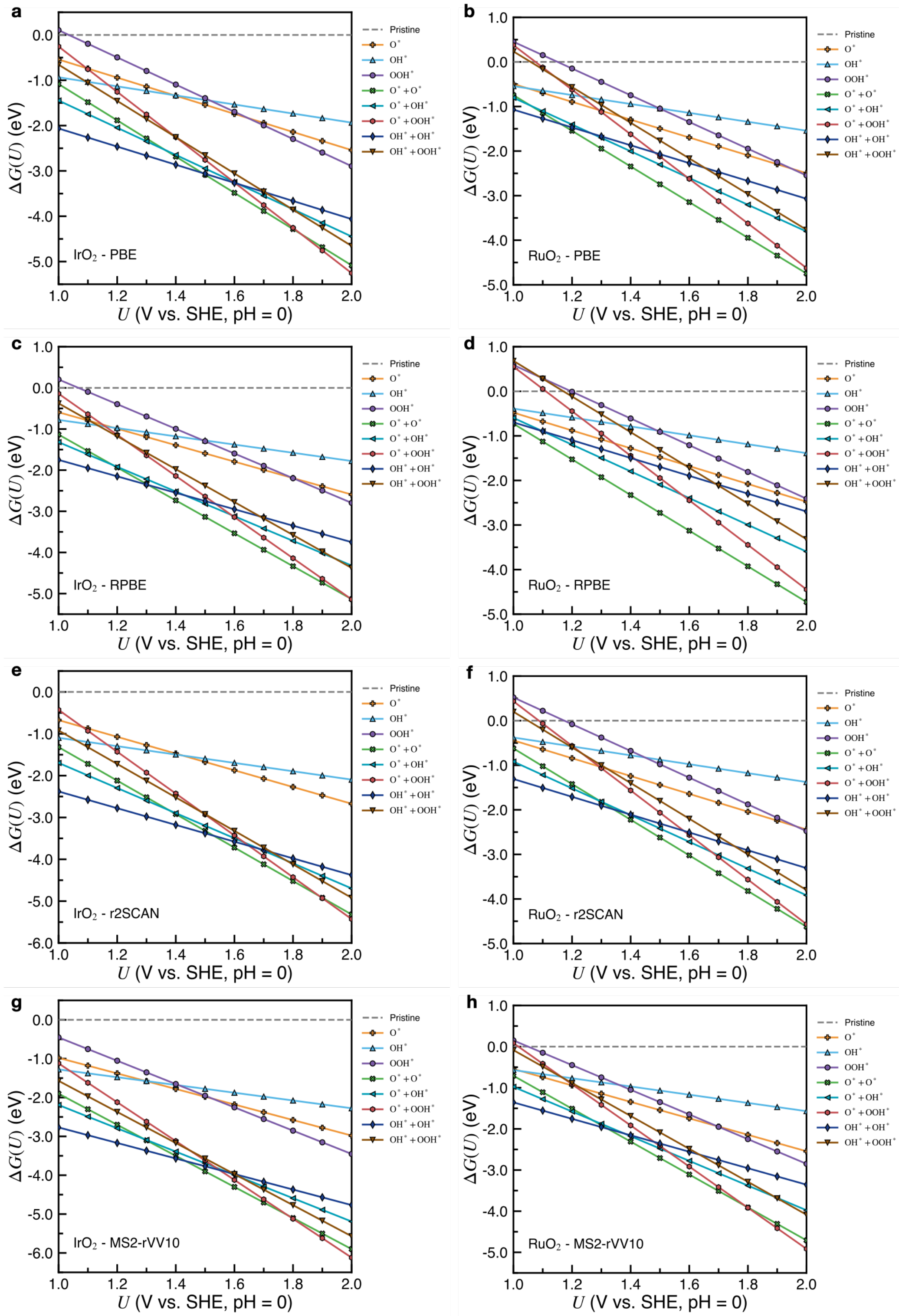

Figure S9. Calculated surface Pourbaix diagrams of IrO_2 (left) and RuO_2 (right) using (a, b) PBE, (c, d) RPBE, (e, f) r2SCAN, and (g, h) MS2-rVV10.

Comparison with the bridge-assisted AEM

In the conventional AEM, the four proton–electron transfer steps proceed through OH*, O*, and OOH* at a coordinatively unsaturated metal site (see Methods). An alternative bridge-assisted AEM has been proposed in which protonation of an adjacent bridging oxygen introduces O* + H_B* and O_2* + H_B* configurations.[9] This mechanism involves the following proton-electron transfer (PCET) steps:

$$H_2O\ (l) + * \rightarrow O* + H_B* + H^+ + e^- \quad (S1)$$
$$O* + H_B* \rightarrow O* + H^+ + e^- \quad (S2)$$
$$O* + H_2O\ (l) \rightarrow O_2* + H_B* + H^+ + e^- \quad (S3)$$
$$O_2* + H_B* \rightarrow * + O_2\ (g) + H^+ + e^- \quad (S4)$$

To assess whether this description affects the conclusions of the present benchmark, we calculated the corresponding free energy, overpotentials and scaling relationships. As shown in Figure S10 and Table S8, the relative stability of OH* and O* + H_B* varies with the XC functional but remains within 0.24 eV, whereas O_2* + H_B* is consistently more stable than OOH* by 0.21–0.51 eV. For all four functionals, the bridge-assisted AEM predicts a larger overpotential for IrO_2 than for RuO_2, thereby reproducing the experimentally observed activity ordering, consistent with previous theoretical studies[9].

The bridge-assisted pathway nevertheless exhibits a distinct, element-dependent scaling behavior. The quantity $\Delta G_{O_2*+H_B*}$-ΔG_{O*+H_B*}, denoted *c*, ranges from 2.57 to 2.91 eV for IrO_2 and from 2.70 to 2.94 eV for RuO_2, consistent with previously reported values of approximately 2.6 eV for Ir and 3.0 eV for Ru.[10] Consequently, the scaling-limited minimum overpotential, $\eta_{min} = \max[c/2, (4.92 - c)/2] - 1.23$, is lower for IrO_2 than for RuO_2 for every functional considered (0.06–0.23 *vs.* 0.12–0.24 V; see Table S8 for the individual values), compared with 0.37 V imposed by the near-universal conventional relation ($c \approx 3.20$ eV). Thus, although the calculated overpotentials of the pristine surfaces correctly predict RuO_2 to be more active, the bridge-assisted scaling relation itself permits a more favorable theoretical activity limit for IrO_2 and therefore does not, by itself, explain the intrinsically higher OER activity of RuO_2.

We further note that selecting the lowest-energy configuration at each oxidation state defines a thermodynamic minimum-energy pathway but does not by itself establish the operative sequence of elementary reaction steps. The bridge-assisted AEM therefore provides a possible alternative description of the OER thermodynamics but is not required to recover the experimental IrO_2/RuO_2 activity ordering, which the conventional AEM also reproduces when the XC functional and spin polarization are treated appropriately.

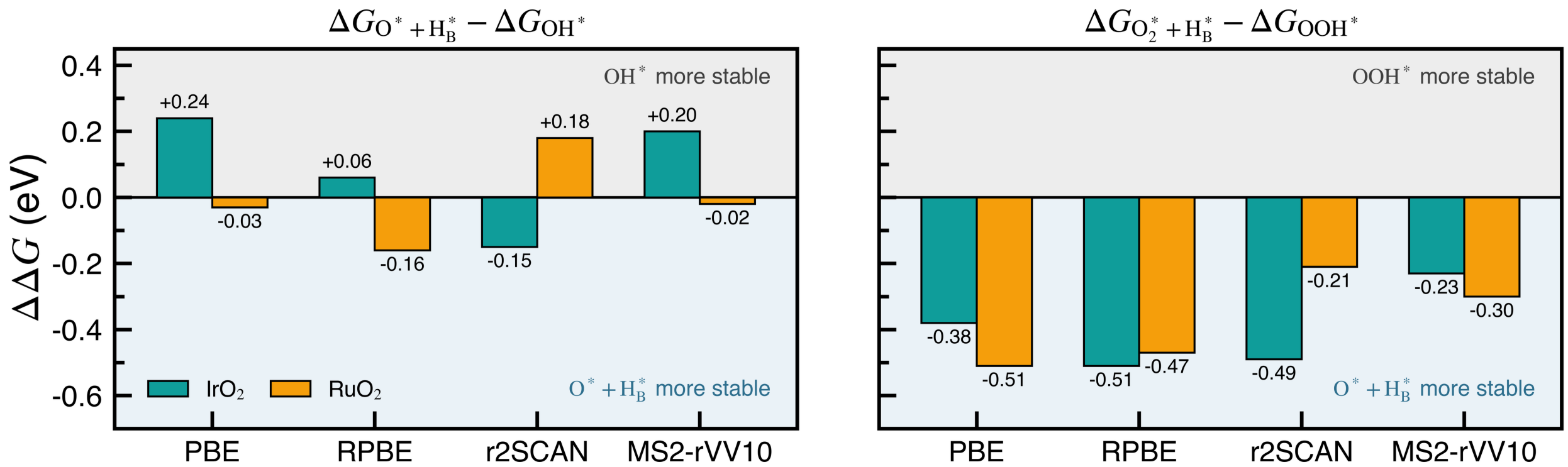

Figure S10. Relative thermodynamic stability of the conventional OH*, OOH* intermediates and the corresponding bridge-H configurations O* + H_B* and O_2* + H_B* on IrO_2 and RuO_2, calculated with spin polarization using the PBE, RPBE, r2SCAN, and MS2-rVV10 functionals. The left panel shows $\Delta\Delta G = \Delta G_{O^*+H_B^*} - \Delta G_{OH^*}$, and the right panel $\Delta\Delta G = \Delta G_{O_2^*+H_B^*} - \Delta G_{OOH^*}$. Negative values indicate that the bridge-protonated configuration is more stable. H_B* denotes hydrogen adsorbed on a neighboring bridging oxygen site.

Table S1. Adsorption free energies of OER intermediates calculated with the PBE, RPBE, r2SCAN, and MS2-rVV10 functionals. "N" and "Y" denote calculations performed without and with spin polarization, respectively. Free energies are in eV.

XC	Materials	Spin-polarization	Δ*G*[OH*]	Δ*G*[O*]	Δ*G*[OOH*]	Δ*G*[OOH*]-Δ*G*[OH*]
PBE	IrO_2	N	0.13	1.80	3.28	3.15
		Y	0.10	1.50	3.29	3.18
	RuO_2	N	0.74	1.75	3.87	3.12
		Y	0.70	1.75	3.88	3.17
RPBE	IrO_2	N	0.33	1.84	3.47	3.15
		Y	0.29	1.47	3.47	3.18
	RuO_2	N	0.96	1.80	4.05	3.08
		Y	0.90	1.76	4.05	3.15
r2SCAN	IrO_2	N	0.08	1.96	3.25	3.17
		Y	-0.02	1.35	3.23	3.25
	RuO_2	N	0.59	1.71	3.77	3.17
		Y	0.53	1.81	3.86	3.33
MS2-rVV10	IrO_2	N	-0.09	1.71	2.90	3.00
		Y	-0.18	1.08	2.87	3.05
	RuO_2	N	0.52	1.70	3.45	2.93
		Y	0.58	1.91	3.64	3.06

Table S2. Magnetic moments of OER adsorbate atoms on the $RuO_2(110)$ and $IrO_2(110)$ surfaces, calculated using four exchange-correlation functionals. Magnetic moments are in μ_B.

XC	Materials	OH*		O*	OOH*		
		O	H		O^1	O^2	H
PBE	IrO_2	0.118	-0.001	0.690	0.147	0.073	0.000
	RuO_2	0.122	0.007	0.126	-0.006	-0.002	0.000
RPBE	IrO_2	0.125	-0.001	0.711	0.192	0.089	0.000
	RuO_2	0.138	0.007	0.132	0.003	0.000	0.000
r2SCAN	IrO_2	0.227	-0.001	0.812	-0.253	-0.092	0.001
	RuO_2	-0.198	-0.010	-0.227	-0.159	-0.052	0.001
MS2-rVV10	IrO_2	0.216	-0.001	0.814	0.238	0.087	0.000
	RuO_2	0.231	0.009	0.029	0.143	0.045	0.000

Note: O^1 and O^2 denote the proximal (surface-bonded) and distal oxygen atoms in OOH*, respectively.

Table S3. Calculated adsorption free energies of O* and OOH*, the crossover value of ΔG_{OOH*} at which $\Delta G_3 = \Delta G_4$, the reaction free energies of the OOH* formation (ΔG_3) and OOH* decomposition (ΔG_4) steps, and the resulting potential-determining step (PDS) for IrO_2 using the PBE, RPBE, r2SCAN, and MS2-rVV10 functionals with spin polarization.

**XCs**	**ΔG_{O*} (eV)**	**Crossover ΔG_{OOH*} (eV)**	**ΔG_{OOH*} (eV)**	**ΔG_3 ($\Delta G_{OOH*} - \Delta G_{O*}$)**	**ΔG_4 (4.92 − ΔG_{OOH*})**	**PDS**
PBE	1.50	3.21	3.29	1.79	1.63	ΔG_3
RPBE	1.47	3.20	3.47	2.00	1.45	ΔG_3
r2SCAN	1.35	3.14	3.23	1.88	1.69	ΔG_3
MS2-rVV10	1.08	3.00	2.87	1.79	2.05	ΔG_4

Table S4. Calculated kinetic barriers for OOH^* formation using CI-NEB at the PBE level under constant-potential and constant-charge conditions.

**Materials**	**E_a (eV)**	
	Constant-potential	Constant-charge
IrO_2	0.52	0.43
RuO_2	0.80	0.74

Table S5. Adsorption free energies of OER intermediates and the corresponding theoretical overpotentials, computed with the PBE functional across different DFT codes. Free energies are in eV.

Codes	Δ*G*[OH*]	Δ*G*[O*]	Δ*G*[OOH*]	Δ*G*[OOH*]-Δ*G*[OH*]	*η* (V)
			**IrO_2**		
VASP	0.13	1.80	3.28	3.15	0.44
QE	0.12	1.75	3.25	3.14	0.44
FHI-aims	0.09	1.73	3.23	3.14	0.46
GPAW	0.09	1.64	3.25	3.16	0.44
			**RuO_2**		
VASP	0.74	1.75	3.87	3.12	0.89
QE	0.75	1.75	3.89	3.14	0.91
FHI-aims	0.75	1.77	3.88	3.13	0.88
GPAW	0.72	1.70	3.87	3.15	0.94

Table S6. Lattice constants of bulk IrO_2 and RuO_2 calculated using four exchange-correlation functionals, along with their deviations from experimental values.

		**PBE**	**RPBE**	**r2SCAN**	**MS2-rVV10**	**Exp.**[11, 12]
	a_0 (Å)	4.541	4.575	4.506	4.486	4.505
IrO_2	c_0 (Å)	3.187	3.203	3.178	3.162	3.159
	Difference (%)	0.90	1.54	0.62	-0.43	-
	a_0 (Å)	4.522	4.562	4.499	4.482	4.492
RuO_2	c_0 (Å)	3.122	3.137	3.117	3.123	3.107
	Difference (%)	0.67	1.55	0.33	0.53	-

Table S7. Free energy corrections applied in reaction free energy calculations. Corrections are in eV

**Species**	$\Delta E_{ZPE} - T\Delta S + \int C_p dT$
OH^*	0.04
O^*	0.33
OOH^*	0.40
H_2O (l)	0
H_2 (g)	-0.04

Table S8. Reaction free energies of the four elementary steps, the scaling constant *c*, the theoretical overpotential η, and the scaling-limited minimum overpotential η_{min} for the conventional and bridge-assisted AEM on IrO_2 and RuO_2, calculated with spin polarization using the PBE, RPBE, r2SCAN, and MS2-rVV10 functionals. Free energies are in eV and overpotentials in V. The scaling constant is $c = \Delta G_{OOH*} - \Delta G_{OH*}$ for the conventional AEM and $c = \Delta G_{O_2* + H_B*} - \Delta G_{O* + H_B*}$ for the bridge-assisted AEM; $\eta_{min} = \max[c/2, (4.92 - c)/2] - 1.23$.

XCs	conventional AEM	ΔG_1	ΔG_2	ΔG_3	ΔG_4	$\Delta G_{OOH*} - \Delta G_{OH*}$	η (V)	η_{min} (V)
PBE	IrO_2	0.10	1.40	1.79	1.63	3.18	0.56	0.36
	RuO_2	0.70	1.05	2.12	1.04	3.17	0.89	0.36
RPBE	IrO_2	0.29	1.18	2.00	1.45	3.18	0.77	0.36
	RuO_2	0.90	0.86	2.29	0.87	3.15	1.06	0.35
R2SCAN	IrO_2	-0.02	1.36	1.89	1.69	3.25	0.66	0.40
	RuO_2	0.53	1.28	2.05	1.06	3.33	0.82	0.44
MS2-rVV10	IrO_2	-0.18	1.26	1.78	2.05	3.05	0.82	0.30
	RuO_2	0.58	1.32	1.73	1.28	3.06	0.50	0.30
	**bridge-assisted AEM**	ΔG_{S1}	ΔG_{S2}	ΔG_{S3}	ΔG_{S4}	$\Delta G_{O_2* + H_B*} - \Delta G_{O* + H_B*}$	η (V)	η_{min} (V)
PBE	IrO_2	0.34	1.16	1.41	2.01	2.57	0.78	0.06
	RuO_2	0.67	1.08	1.62	1.55	2.70	0.39	0.12
RPBE	IrO_2	0.35	1.11	1.49	1.96	2.61	0.73	0.08
	RuO_2	0.74	1.02	1.82	1.34	2.84	0.59	0.19
R2SCAN	IrO_2	-0.17	1.51	1.39	2.18	2.91	0.95	0.23
	RuO_2	0.71	1.10	1.84	1.27	2.94	0.61	0.24
MS2-rVV10	IrO_2	0.02	1.06	1.56	2.28	2.62	1.05	0.08
	RuO_2	0.56	1.35	1.44	1.58	2.78	0.35	0.16

Reference

1. V. Blum, R. Gehrke, F. Hanke, P. Havu, V. Havu, X. Ren, K. Reuter and M. Scheffler, Computer Physics Communications, 2009, 180, 2175-2196.
2. J. Enkovaara, C. Rostgaard, J. J. Mortensen, J. Chen, M. Dułak, L. Ferrighi, J. Gavnholt, C. Glinsvad, V. Haikola and H. A. Hansen, Journal of physics: Condensed matter, 2010, 22, 253202.
3. P. Giannozzi, S. Baroni, N. Bonini, M. Calandra, R. Car, C. Cavazzoni, D. Ceresoli, G. L. Chiarotti, M. Cococcioni and I. Dabo, Journal of physics: Condensed matter, 2009, 21, 395502.
4. J. P. Perdew, K. Burke and M. Ernzerhof, Physical review letters, 1996, 77, 3865.
5. G. Prandini, A. Marrazzo, I. E. Castelli, N. Mounet and N. Marzari, npj Computational Materials, 2018, 4, 72.
6. A. Laio and M. Parrinello, Proceedings of the national academy of sciences, 2002, 99, 12562-12566.
7. M. Iannuzzi, A. Laio and M. Parrinello, Physical review letters, 2003, 90, 238302.
8. Q. Liang, A. Bieberle-Hütter and G. Brocks, The Journal of Physical Chemistry C, 2022, 126, 1337-1345.
9. S. Divanis, A. M. Frandsen, T. Kutlusoy and J. Rossmeisl, Physical Chemistry Chemical Physics, 2021, 23, 19141-19145.
10. K. L. Svane and J. Rossmeisl, Angewandte Chemie, 2022, 134, e202201146.
11. A. A. Bolzan, C. Fong, B. J. Kennedy and C. J. Howard, Structural Science, 1997, 53, 373-380.
12. C.-E. Boman, J. Danielsen, A. T. Haaland, B. Jerslev, C. E. Schäffer, E. Sunde and N. A. Sörensen, Acta Chemica Scandinavica, 1970, 24, 116-122.
